# Supplementary material for: Machine Learning Based Analysis of Relations between Antigen Expression and Genetic Aberrations in Childhood B-Cell Precursor Acute Lymphoblastic Leukaemia
Source: J Clin Med. 2022 Apr 19;11(9):2281. doi: 10.3390/jcm11092281 (PMC9100578; doi:10.3390/jcm11092281)
Supplement: Supplementary file 1 [file jcm-11-02281-s001.zip › supplementary_material.html]

Machine learning based analysis of relations between antigen expression and genetic aberrations in childhood acute lymphoblastic leukaemia


# Machine learning based analysis of relations between antigen expression and genetic aberrations in childhood acute lymphoblastic leukaemia

#### Supplementary material

## Introduction

- 5 decision problems: t(12;21)/ETV6-RUNX1, t(v;11q23)/KMT2A, hyperdiploidy, no aberration (examples without any aberration), t(12;21)/ETV6-RUNX1 vs. hyperdiploidy
- 17 attributes: CD34, CD45, CD66c, CD10, CD38, CD20, CyIgM, CD33, TdT, CD13, CD22, CD24, CD9, CD15+CD65, CD123, CD81, NG2
- number of observations in the original data set: 818
- number of observations after removing rows with 100% missing in X: 695
- number of observations after removing rows with 100% missing in Y: 670
- number of observations after removing rows with more than 50% missing values in X and 0/NA in Y: 606

### Descriptive statistics

### Counts

| decision problem | 0 | 1 | NA |
| --- | --- | --- | --- |
| t(12;21)/ETV6-RUNX1 | 422 | 52 | 132 |
| t(v;11q23)/KMT2A | 588 | 17 | 1 |
| hyperdiploidy | 378 | 124 | 104 |
| no aberration (examples without any aberration) | 190 | 257 | 159 |
| t(12;21)/ETV6-RUNX1 vs. hyperdiploidy | 121 | 52 | 433 |

### Missing data

#### X variables

##### Y variables

## Predictive analysis

Utilize Recursive Partitioning and Regression Trees from rpart package and methods (especially Gradient Boosting Machine) from h2o package in 10 x 5CV mode.

In model selection we have used:

- GLM - Generalized Linear Model,
- DRF - Distributed Random Forest,
- GBM - Gradient Boosting Machine,
- AML - Auto Machine Learning - Stacked Ensemble models,

with different strategies of hyperparameters optimization:

- default - default hyperparameters,
- best-tuning - optimization on full dataset,
- fold-tuning - optimization on each CV fold.

### Model selection - train

Model selection was conducted based on AUC measure.

#### t(12;21)/ETV6-RUNX1

| algorithm | mean | sd | min | max |
| --- | --- | --- | --- | --- |
| GBM\_default | 0.9998850 | 0.0001175 | 0.9996946 | 1.0000000 |
| GBM\_fold\_tuning | 0.9806674 | 0.0257683 | 0.9251492 | 1.0000000 |
| GBM\_best\_tuning | 0.9153129 | 0.0095509 | 0.8976127 | 0.9274916 |
| GLM\_default | 0.9090318 | 0.0121553 | 0.8942183 | 0.9370389 |
| AML\_default | 0.8639371 | 0.1157939 | 0.6639242 | 1.0000000 |
| DRF\_default | 0.7327323 | 0.0266094 | 0.6736740 | 0.7596203 |
| DRF\_best\_tuning | 0.6784206 | 0.0263560 | 0.6396281 | 0.7133570 |
| DRF\_fold\_tuning | NaN | NA | NaN | NaN |

#### t(v;11q23)/KMT2A

| algorithm | mean | sd | min | max |
| --- | --- | --- | --- | --- |
| GBM\_default | 1.0000000 | 0.0000000 | 1.0000000 | 1.0000000 |
| GLM\_default | 0.9998276 | 0.0005452 | 0.9982759 | 1.0000000 |
| GBM\_best\_tuning | 0.9996222 | 0.0003067 | 0.9988623 | 1.0000000 |
| GBM\_fold\_tuning | 0.9982977 | 0.0026919 | 0.9924475 | 1.0000000 |
| AML\_default | 0.9613082 | 0.0491434 | 0.8906047 | 1.0000000 |
| DRF\_default | 0.8649069 | 0.0465267 | 0.7844407 | 0.9211604 |
| DRF\_best\_tuning | NaN | NA | NaN | NaN |
| DRF\_fold\_tuning | NaN | NA | NaN | NaN |

#### hyperdiploidy

| algorithm | mean | sd | min | max |
| --- | --- | --- | --- | --- |
| GBM\_default | 0.9975811 | 0.0009971 | 0.9958310 | 0.9991378 |
| GBM\_best\_tuning | 0.9909131 | 0.0017519 | 0.9874693 | 0.9935398 |
| GBM\_fold\_tuning | 0.9446475 | 0.0470378 | 0.8562849 | 0.9999882 |
| AML\_default | 0.8907420 | 0.0851610 | 0.8005437 | 1.0000000 |
| GLM\_default | 0.8228283 | 0.0153270 | 0.8060114 | 0.8507614 |
| DRF\_best\_tuning | 0.7910353 | 0.0136755 | 0.7691859 | 0.8063778 |
| DRF\_default | 0.7699460 | 0.0122580 | 0.7481930 | 0.7845465 |
| DRF\_fold\_tuning | NaN | NA | NaN | NaN |

### Model selection - test

Model selection was conducted based on AUC measure.

#### t(12;21)/ETV6-RUNX1

| algorithm | mean | sd | min | max |
| --- | --- | --- | --- | --- |
| GLM\_default | 0.8837449 | 0.1035621 | 0.6862745 | 1.0000000 |
| GBM\_fold\_tuning | 0.8785816 | 0.0843375 | 0.7260870 | 0.9826087 |
| DRF\_fold\_tuning | 0.8753623 | 0.0960450 | 0.6565217 | 0.9826087 |
| GBM\_best\_tuning | 0.7946099 | 0.1220147 | 0.5391304 | 0.9130435 |
| GBM\_default | 0.7812797 | 0.1205425 | 0.6130435 | 0.9217391 |
| DRF\_best\_tuning | 0.7804780 | 0.1356822 | 0.5695652 | 0.9478261 |
| DRF\_default | 0.7695436 | 0.1322566 | 0.5869565 | 0.9347826 |
| AML\_default | 0.7164570 | 0.1473597 | 0.4652174 | 0.9130435 |

#### t(v;11q23)/KMT2A

| algorithm | mean | sd | min | max |
| --- | --- | --- | --- | --- |
| DRF\_fold\_tuning | 0.9471970 | 0.1327415 | 0.5719697 | 1 |
| GBM\_fold\_tuning | 0.9243939 | 0.1176841 | 0.6439394 | 1 |
| DRF\_default | 0.8877098 | 0.2312182 | 0.2462121 | 1 |
| GBM\_default | 0.8393124 | 0.1905149 | 0.4621212 | 1 |
| DRF\_best\_tuning | 0.8103963 | 0.3004466 | 0.2424242 | 1 |
| AML\_default | 0.8029254 | 0.1935169 | 0.4538462 | 1 |
| GBM\_best\_tuning | 0.7700466 | 0.3098041 | 0.0615385 | 1 |
| GLM\_default | 0.3857143 | 0.4996597 | 0.0000000 | 1 |

#### hyperdiploidy

| algorithm | mean | sd | min | max |
| --- | --- | --- | --- | --- |
| GBM\_fold\_tuning | 0.8458791 | 0.0731504 | 0.7179487 | 0.9194139 |
| DRF\_fold\_tuning | 0.8446276 | 0.0634158 | 0.7321429 | 0.9230769 |
| DRF\_best\_tuning | 0.7963217 | 0.0706159 | 0.6865079 | 0.8974359 |
| GBM\_best\_tuning | 0.7925061 | 0.0744698 | 0.6488095 | 0.8681319 |
| GBM\_default | 0.7904304 | 0.0753719 | 0.6263736 | 0.8690476 |
| DRF\_default | 0.7855998 | 0.0525794 | 0.7216117 | 0.8720238 |
| GLM\_default | 0.7728035 | 0.1177852 | 0.5933333 | 0.9523810 |
| AML\_default | 0.7630800 | 0.0980562 | 0.6011905 | 0.8901099 |

### Prediction RPART

#### t(12;21)/ETV6-RUNX1

```
          Reference
Prediction     0     1
         0 405.9  45.8
         1  16.1   6.2
```

| stat | mean | sd | min | max |
| --- | --- | --- | --- | --- |
| Accuracy | 0.8694 | 0.0058 | 0.8629 | 0.8797 |
| Balanced Accuracy | 0.5405 | 0.0201 | 0.5111 | 0.5700 |
| F1 | 0.1653 | 0.0524 | 0.0857 | 0.2400 |
| Neg Pred Value | 0.8986 | 0.0042 | 0.8925 | 0.9047 |
| Pos Pred Value | 0.2733 | 0.0713 | 0.1667 | 0.3913 |
| Precision | 0.2733 | 0.0713 | 0.1667 | 0.3913 |
| Sensitivity | 0.1192 | 0.0413 | 0.0577 | 0.1731 |
| Specificity | 0.9618 | 0.0057 | 0.9526 | 0.9692 |

#### t(v;11q23)/KMT2A

```
          Reference
Prediction   0   1
         0 585   8
         1   3   9
```

| stat | mean | sd | min | max |
| --- | --- | --- | --- | --- |
| Accuracy | 0.9818 | 0 | 0.9818 | 0.9818 |
| Balanced Accuracy | 0.7622 | 0 | 0.7622 | 0.7622 |
| F1 | 0.6207 | 0 | 0.6207 | 0.6207 |
| Neg Pred Value | 0.9865 | 0 | 0.9865 | 0.9865 |
| Pos Pred Value | 0.7500 | 0 | 0.7500 | 0.7500 |
| Precision | 0.7500 | 0 | 0.7500 | 0.7500 |
| Sensitivity | 0.5294 | 0 | 0.5294 | 0.5294 |
| Specificity | 0.9949 | 0 | 0.9949 | 0.9949 |

#### hyperdiploidy

```
          Reference
Prediction     0     1
         0 334.1  76.2
         1  43.9  47.8
```

| stat | mean | sd | min | max |
| --- | --- | --- | --- | --- |
| Accuracy | 0.7608 | 0.0126 | 0.7410 | 0.7869 |
| Balanced Accuracy | 0.6347 | 0.0177 | 0.6057 | 0.6715 |
| F1 | 0.4427 | 0.0319 | 0.3883 | 0.5069 |
| Neg Pred Value | 0.8144 | 0.0086 | 0.8000 | 0.8313 |
| Pos Pred Value | 0.5223 | 0.0348 | 0.4694 | 0.5914 |
| Precision | 0.5223 | 0.0348 | 0.4694 | 0.5914 |
| Sensitivity | 0.3855 | 0.0374 | 0.3226 | 0.4435 |
| Specificity | 0.8839 | 0.0164 | 0.8598 | 0.9074 |

#### no aberration (examples without any aberration)

```
          Reference
Prediction     0     1
         0  95.2  75.5
         1  94.8 181.5
```

| stat | mean | sd | min | max |
| --- | --- | --- | --- | --- |
| Accuracy | 0.6190 | 0.0146 | 0.5951 | 0.6376 |
| Balanced Accuracy | 0.6036 | 0.0142 | 0.5779 | 0.6196 |
| F1 | 0.6805 | 0.0159 | 0.6526 | 0.7011 |
| Neg Pred Value | 0.5583 | 0.0208 | 0.5246 | 0.5864 |
| Pos Pred Value | 0.6570 | 0.0116 | 0.6357 | 0.6718 |
| Precision | 0.6570 | 0.0116 | 0.6357 | 0.6718 |
| Sensitivity | 0.7062 | 0.0291 | 0.6615 | 0.7549 |
| Specificity | 0.5011 | 0.0295 | 0.4474 | 0.5526 |

#### t(12;21)/ETV6-RUNX1 vs. hyperdiploidy

```
          Reference
Prediction     0     1
         0 106.3  26.0
         1  14.7  26.0
```

| stat | mean | sd | min | max |
| --- | --- | --- | --- | --- |
| Accuracy | 0.7647 | 0.0244 | 0.7283 | 0.7919 |
| Balanced Accuracy | 0.6893 | 0.0330 | 0.6358 | 0.7333 |
| F1 | 0.5593 | 0.0527 | 0.4719 | 0.6275 |
| Neg Pred Value | 0.8041 | 0.0212 | 0.7721 | 0.8374 |
| Pos Pred Value | 0.6432 | 0.0658 | 0.5641 | 0.7586 |
| Precision | 0.6432 | 0.0658 | 0.5641 | 0.7586 |
| Sensitivity | 0.5000 | 0.0708 | 0.4038 | 0.6154 |
| Specificity | 0.8785 | 0.0317 | 0.8430 | 0.9421 |

### Prediction GBM

#### t(12;21)/ETV6-RUNX1

```
          Reference
Prediction     0     1
         0 373.2  16.6
         1  48.8  35.4
```

| stat | mean | sd | min | max |
| --- | --- | --- | --- | --- |
| Accuracy | 0.8620 | 0.0229 | 0.8186 | 0.8924 |
| Balanced Accuracy | 0.7826 | 0.0176 | 0.7423 | 0.8038 |
| F1 | 0.5219 | 0.0287 | 0.4819 | 0.5714 |
| Neg Pred Value | 0.9576 | 0.0062 | 0.9457 | 0.9667 |
| Pos Pred Value | 0.4278 | 0.0473 | 0.3509 | 0.5075 |
| Precision | 0.4278 | 0.0473 | 0.3509 | 0.5075 |
| Sensitivity | 0.6808 | 0.0575 | 0.5769 | 0.7692 |
| Specificity | 0.8844 | 0.0314 | 0.8246 | 0.9218 |

#### t(v;11q23)/KMT2A

```
          Reference
Prediction     0     1
         0 581.4   6.9
         1   6.6  10.1
```

| stat | mean | sd | min | max |
| --- | --- | --- | --- | --- |
| Accuracy | 0.9777 | 0.0102 | 0.9504 | 0.9851 |
| Balanced Accuracy | 0.7914 | 0.0362 | 0.7336 | 0.8487 |
| F1 | 0.6110 | 0.0845 | 0.4231 | 0.7059 |
| Neg Pred Value | 0.9883 | 0.0021 | 0.9849 | 0.9915 |
| Pos Pred Value | 0.6599 | 0.1544 | 0.3143 | 0.8333 |
| Precision | 0.6599 | 0.1544 | 0.3143 | 0.8333 |
| Sensitivity | 0.5941 | 0.0757 | 0.4706 | 0.7059 |
| Specificity | 0.9888 | 0.0111 | 0.9592 | 0.9966 |

#### hyperdiploidy

```
          Reference
Prediction     0     1
         0 307.5  26.7
         1  70.5  97.3
```

| stat | mean | sd | min | max |
| --- | --- | --- | --- | --- |
| Accuracy | 0.8064 | 0.0097 | 0.7829 | 0.8167 |
| Balanced Accuracy | 0.7991 | 0.0123 | 0.7770 | 0.8135 |
| F1 | 0.6668 | 0.0121 | 0.6431 | 0.6777 |
| Neg Pred Value | 0.9205 | 0.0123 | 0.9008 | 0.9353 |
| Pos Pred Value | 0.5810 | 0.0184 | 0.5389 | 0.6013 |
| Precision | 0.5810 | 0.0184 | 0.5389 | 0.6013 |
| Sensitivity | 0.7847 | 0.0410 | 0.7177 | 0.8387 |
| Specificity | 0.8135 | 0.0225 | 0.7646 | 0.8413 |

#### no aberration (examples without any aberration)

```
          Reference
Prediction     0     1
         0  78.7  19.1
         1 111.3 237.9
```

| stat | mean | sd | min | max |
| --- | --- | --- | --- | --- |
| Accuracy | 0.7083 | 0.0118 | 0.6801 | 0.7248 |
| Balanced Accuracy | 0.6699 | 0.0164 | 0.6305 | 0.6914 |
| F1 | 0.7850 | 0.0047 | 0.7755 | 0.7926 |
| Neg Pred Value | 0.8071 | 0.0215 | 0.7692 | 0.8507 |
| Pos Pred Value | 0.6817 | 0.0137 | 0.6500 | 0.6994 |
| Precision | 0.6817 | 0.0137 | 0.6500 | 0.6994 |
| Sensitivity | 0.9257 | 0.0171 | 0.8949 | 0.9611 |
| Specificity | 0.4142 | 0.0482 | 0.3000 | 0.4737 |

#### t(12;21)/ETV6-RUNX1 vs. hyperdiploidy

```
          Reference
Prediction     0     1
         0 110.8   9.7
         1  10.2  42.3
```

| stat | mean | sd | min | max |
| --- | --- | --- | --- | --- |
| Accuracy | 0.8850 | 0.0160 | 0.8555 | 0.9017 |
| Balanced Accuracy | 0.8646 | 0.0092 | 0.8528 | 0.8789 |
| F1 | 0.8102 | 0.0143 | 0.7788 | 0.8283 |
| Neg Pred Value | 0.9206 | 0.0187 | 0.8992 | 0.9619 |
| Pos Pred Value | 0.8147 | 0.0677 | 0.7059 | 0.9070 |
| Precision | 0.8147 | 0.0677 | 0.7059 | 0.9070 |
| Sensitivity | 0.8135 | 0.0529 | 0.7500 | 0.9231 |
| Specificity | 0.9157 | 0.0432 | 0.8347 | 0.9669 |

### Variable importance

Average rank based on training folds in 5 cross validation (repeated 10 times) based on tree split

#### t(12;21)/ETV6-RUNX1

#### t(v;11q23)/KMT2A

#### hyperdiploidy

#### no aberration (examples without any aberration)

#### t(12;21)/ETV6-RUNX1 vs. hyperdiploidy

## Descriptive analysis

### RPART decision trees

#### t(12;21)/ETV6-RUNX1

```
Confusion Matrix and Statistics

          Reference
Prediction   0   1
         0 420  14
         1   2  38
                                          
               Accuracy : 0.9662          
                 95% CI : (0.9458, 0.9806)
    No Information Rate : 0.8903          
    P-Value [Acc > NIR] : 1.125e-09       
                                          
                  Kappa : 0.8077          
                                          
 Mcnemar's Test P-Value : 0.00596         
                                          
            Sensitivity : 0.73077         
            Specificity : 0.99526         
         Pos Pred Value : 0.95000         
         Neg Pred Value : 0.96774         
              Precision : 0.95000         
                 Recall : 0.73077         
                     F1 : 0.82609         
             Prevalence : 0.10970         
         Detection Rate : 0.08017         
   Detection Prevalence : 0.08439         
      Balanced Accuracy : 0.86301         
                                          
       'Positive' Class : 1
```

|  | Variable importance |
| --- | --- |
| CD10 | 25.1423989 |
| CD24 | 21.9549454 |
| CD34 | 11.3961721 |
| CD38 | 10.1428889 |
| CD45 | 8.7825103 |
| TdT | 7.4577273 |
| CyIgM | 6.7315470 |
| CD66c | 6.6255950 |
| CD13 | 5.2358929 |
| CD33 | 4.7591406 |
| CD81 | 3.8538350 |
| CD22 | 3.4798346 |
| CD123 | 1.7142857 |
| CD20 | 0.1669621 |
| CD9 | 0.1037234 |

| probability: primary class | x | x\_2 | x\_3 | x\_4 | x\_5 | x\_6 | x\_7 | x\_8 | x\_9 | x\_10 | x\_11 | x\_12 | x\_13 | x\_14 | x\_15 | x\_16 | x\_17 | x\_18 | x\_19 | x\_20 | x\_21 | x\_22 | x\_23 | x\_24 | x\_25 | x\_26 | x\_27 | x\_28 | x\_29 | x\_30 | x\_31 | x\_32 | x\_33 | x\_34 | x\_35 | x\_36 | x\_37 | x\_38 | x\_39 | x\_40 | x\_41 | x\_42 | x\_43 | x\_44 | x\_45 | x\_46 | x\_47 | x\_48 | x\_49 | x\_50 | x\_51 | x\_52 | x\_53 | x\_54 | x\_55 | x\_56 | x\_57 | x\_58 | x\_59 | x\_60 | x\_61 | x\_62 | x\_63 | x\_64 | cover | cover\_cumulative | rule\_no |
| --- | --- | --- | --- | --- | --- | --- | --- | --- | --- | --- | --- | --- | --- | --- | --- | --- | --- | --- | --- | --- | --- | --- | --- | --- | --- | --- | --- | --- | --- | --- | --- | --- | --- | --- | --- | --- | --- | --- | --- | --- | --- | --- | --- | --- | --- | --- | --- | --- | --- | --- | --- | --- | --- | --- | --- | --- | --- | --- | --- | --- | --- | --- | --- | --- | --- | --- | --- |
| 0.04 | when | CD66c | >= |  |  | 1 | & | CD10 | is | 25 | to | 98 | & | CD45 | < | 4 | & | CD24 | < | 101 |  |  | & | CD13 | < | 24 | & | CD38 | < | 20 | & | CD22 | >= |  |  | 2 |  |  |  |  |  |  |  |  |  |  |  |  |  |  |  |  |  |  |  |  |  |  |  |  |  |  |  |  | 30% | 30% | 1 |
| 0.00 | when | CD66c | >= |  |  | 1 | & | CD10 | < | 25 |  |  |  |  |  |  | & | CD24 | < | 101 |  |  | & | CD13 | < | 24 | & | CD38 | < | 20 |  |  |  |  |  |  |  |  |  |  |  |  |  |  |  |  |  |  |  |  |  |  |  |  |  |  | & | CD9 | >= | 2 |  |  |  |  | 27% | 57% | 2 |
| 0.00 | when | CD66c | < | 1 |  |  |  |  |  |  |  |  | & | CD45 | >= | 2 |  |  |  |  |  |  |  |  |  |  |  |  |  |  | & | CD22 | >= |  |  | 3 |  |  |  |  |  |  |  |  |  |  |  |  |  |  |  |  |  |  |  |  |  |  |  |  |  |  |  |  | 6% | 63% | 3 |
| 0.00 | when | CD66c | < | 1 |  |  | & | CD10 | < | 16 |  |  | & | CD45 | >= | 2 |  |  |  |  |  |  |  |  |  |  |  |  |  |  | & | CD22 | < | 3 |  |  |  |  |  |  |  |  |  |  |  |  |  |  | & | TdT | < | 5 |  |  |  |  |  |  |  |  |  |  |  |  | 5% | 68% | 4 |
| 0.00 | when | CD66c | >= |  |  | 1 | & | CD10 | < | 25 |  |  | & | CD45 | >= | 2 | & | CD24 | < | 101 |  |  | & | CD13 | < | 24 | & | CD38 | < | 20 |  |  |  |  |  |  |  |  |  |  |  |  |  |  |  |  |  |  |  |  |  |  |  |  |  |  | & | CD9 | < | 2 |  |  |  |  | 3% | 71% | 5 |
| 0.00 | when | CD66c | < | 1 |  |  | & | CD10 | < | 36 |  |  | & | CD45 | < | 2 | & | CD24 | >= |  |  | 38 |  |  |  |  |  |  |  |  |  |  |  |  |  |  |  |  |  |  |  |  |  |  |  |  |  |  |  |  |  |  |  |  |  |  |  |  |  |  |  |  |  |  | 3% | 74% | 6 |
| 0.33 | when | CD66c | < | 1 |  |  | & | CD10 | < | 54 |  |  | & | CD45 | < | 2 | & | CD24 | < | 38 |  |  |  |  |  |  | & | CD38 | < | 2 |  |  |  |  |  |  | & | CD34 | >= | 4 |  |  |  |  | & | CD33 | >= | 1 |  |  |  |  |  |  |  |  |  |  |  |  |  |  |  |  | 3% | 77% | 7 |
| 0.00 | when | CD66c | < | 1 |  |  | & | CD10 | >= |  |  | 17 | & | CD45 | >= | 2 |  |  |  |  |  |  |  |  |  |  |  |  |  |  | & | CD22 | < | 2 |  |  |  |  |  |  |  |  |  |  |  |  |  |  |  |  |  |  |  |  |  |  |  |  |  |  | & | CD123 | < | 1 | 2% | 79% | 8 |
| 0.00 | when | CD66c | >= |  |  | 1 | & | CD10 | < | 98 |  |  |  |  |  |  | & | CD24 | >= |  |  | 101 | & | CD13 | < | 24 | & | CD38 | < | 20 |  |  |  |  |  |  |  |  |  |  | & | CyIgM | < | 5 |  |  |  |  |  |  |  |  |  |  |  |  |  |  |  |  |  |  |  |  | 1% | 80% | 9 |
| 0.00 | when | CD66c | >= |  |  | 1 | & | CD10 | >= |  |  | 98 |  |  |  |  |  |  |  |  |  |  |  |  |  |  | & | CD38 | >= | 2 |  |  |  |  |  |  |  |  |  |  |  |  |  |  | & | CD33 | < | 1 |  |  |  |  |  |  |  |  |  |  |  |  |  |  |  |  | 1% | 81% | 10 |
| 0.00 | when | CD66c | >= |  |  | 1 | & | CD10 | >= |  |  | 98 |  |  |  |  | & | CD24 | >= |  |  | 32 |  |  |  |  | & | CD38 | < | 2 |  |  |  |  |  |  |  |  |  |  |  |  |  |  | & | CD33 | < | 1 |  |  |  |  |  |  |  |  |  |  |  |  |  |  |  |  | 1% | 82% | 11 |
| 0.00 | when | CD66c | < | 1 |  |  | & | CD10 | < | 16 |  |  | & | CD45 | >= | 2 |  |  |  |  |  |  |  |  |  |  |  |  |  |  | & | CD22 | < | 3 |  |  |  |  |  |  |  |  |  |  |  |  |  |  | & | TdT | >= | 5 | & | CD81 | >= | 3 |  |  |  |  |  |  |  |  | 1% | 83% | 12 |
| 0.00 | when | CD66c | < | 1 |  |  | & | CD10 | is | 50 | to | 71 | & | CD45 | >= | 2 |  |  |  |  |  |  |  |  |  |  |  |  |  |  | & | CD22 | is | 2 | to | 3 |  |  |  |  |  |  |  |  |  |  |  |  |  |  |  |  |  |  |  |  |  |  |  |  |  |  |  |  | 1% | 84% | 13 |
| 0.00 | when | CD66c | < | 1 |  |  | & | CD10 | >= |  |  | 36 | & | CD45 | < | 2 | & | CD24 | >= |  |  | 58 | & | CD13 | < | 4 |  |  |  |  |  |  |  |  |  |  |  |  |  |  |  |  |  |  |  |  |  |  |  |  |  |  |  |  |  |  |  |  |  |  |  |  |  |  | 1% | 85% | 14 |
| 0.00 | when | CD66c | < | 1 |  |  | & | CD10 | >= |  |  | 64 | & | CD45 | < | 2 | & | CD24 | < | 38 |  |  |  |  |  |  |  |  |  |  |  |  |  |  |  |  | & | CD34 | >= | 4 |  |  |  |  |  |  |  |  |  |  |  |  |  |  |  |  |  |  |  |  |  |  |  |  | 1% | 86% | 15 |
| 0.00 | when | CD66c | < | 1 |  |  | & | CD10 | < | 54 |  |  | & | CD45 | < | 2 | & | CD24 | < | 38 |  |  |  |  |  |  |  |  |  |  |  |  |  |  |  |  | & | CD34 | >= | 4 |  |  |  |  | & | CD33 | < | 1 |  |  |  |  |  |  |  |  |  |  |  |  |  |  |  |  | 1% | 87% | 16 |
| 0.00 | when | CD66c | < | 1 |  |  |  |  |  |  |  |  | & | CD45 | < | 2 | & | CD24 | is | 26 | to | 35 |  |  |  |  |  |  |  |  |  |  |  |  |  |  | & | CD34 | < | 4 | & | CyIgM | < | 3 |  |  |  |  |  |  |  |  |  |  |  |  |  |  |  |  |  |  |  |  | 1% | 88% | 17 |
| 0.17 | when | CD66c | >= |  |  | 4 | & | CD10 | is | 25 | to | 98 |  |  |  |  | & | CD24 | < | 101 |  |  | & | CD13 | < | 24 | & | CD38 | < | 20 | & | CD22 | < | 2 |  |  |  |  |  |  |  |  |  |  |  |  |  |  |  |  |  |  |  |  |  |  |  |  |  |  |  |  |  |  | 1% | 89% | 18 |
| 0.33 | when | CD66c | >= |  |  | 1 | & | CD10 | < | 25 |  |  | & | CD45 | < | 2 | & | CD24 | < | 101 |  |  | & | CD13 | < | 24 | & | CD38 | < | 20 |  |  |  |  |  |  |  |  |  |  |  |  |  |  |  |  |  |  |  |  |  |  |  |  |  |  | & | CD9 | < | 2 |  |  |  |  | 1% | 90% | 19 |
| 0.67 | when | CD66c | < | 1 |  |  | & | CD10 | < | 95 |  |  | & | CD45 | < | 2 | & | CD24 | < | 26 |  |  | & | CD13 | >= | 3 |  |  |  |  |  |  |  |  |  |  | & | CD34 | < | 4 |  |  |  |  |  |  |  |  |  |  |  |  |  |  |  |  |  |  |  |  |  |  |  |  | 1% | 91% | 20 |
| 1.00 | when | CD66c | >= |  |  | 1 | & | CD10 | >= |  |  | 98 |  |  |  |  | & | CD24 | < | 32 |  |  |  |  |  |  | & | CD38 | < | 2 |  |  |  |  |  |  |  |  |  |  |  |  |  |  | & | CD33 | < | 1 |  |  |  |  |  |  |  |  |  |  |  |  |  |  |  |  | 1% | 92% | 21 |
| 1.00 | when | CD66c | < | 1 |  |  | & | CD10 | >= |  |  | 36 | & | CD45 | < | 2 | & | CD24 | is | 38 | to | 58 | & | CD13 | < | 4 |  |  |  |  |  |  |  |  |  |  |  |  |  |  | & | CyIgM | < | 5 |  |  |  |  | & | TdT | < | 12 |  |  |  |  |  |  |  |  |  |  |  |  | 1% | 93% | 22 |
| 1.00 | when | CD66c | < | 1 |  |  | & | CD10 | is | 54 | to | 64 | & | CD45 | < | 2 | & | CD24 | < | 38 |  |  |  |  |  |  |  |  |  |  |  |  |  |  |  |  | & | CD34 | >= | 4 |  |  |  |  |  |  |  |  |  |  |  |  |  |  |  |  |  |  |  |  |  |  |  |  | 1% | 94% | 23 |
| 1.00 | when | CD66c | < | 1 |  |  | & | CD10 | < | 95 |  |  | & | CD45 | < | 2 | & | CD24 | < | 26 |  |  | & | CD13 | < | 3 |  |  |  |  |  |  |  |  |  |  | & | CD34 | < | 4 |  |  |  |  |  |  |  |  |  |  |  |  |  |  |  |  |  |  |  |  |  |  |  |  | 1% | 95% | 24 |
| 1.00 | when | CD66c | < | 1 |  |  |  |  |  |  |  |  | & | CD45 | < | 2 | & | CD24 | is | 35 | to | 38 |  |  |  |  |  |  |  |  |  |  |  |  |  |  | & | CD34 | < | 4 |  |  |  |  |  |  |  |  |  |  |  |  |  |  |  |  |  |  |  |  |  |  |  |  | 1% | 96% | 25 |
| 0.00 | when | CD66c | < | 1 |  |  | & | CD10 | is | 16 | to | 50 | & | CD45 | >= | 2 |  |  |  |  |  |  |  |  |  |  |  |  |  |  | & | CD22 | is | 2 | to | 3 |  |  |  |  |  |  |  |  |  |  |  |  |  |  |  |  | & | CD81 | < | 3 |  |  |  |  |  |  |  |  | 0% | 96% | 26 |
| 0.00 | when | CD66c | < | 1 |  |  | & | CD10 | >= |  |  | 36 | & | CD45 | < | 2 | & | CD24 | >= |  |  | 38 | & | CD13 | >= | 4 |  |  |  |  |  |  |  |  |  |  |  |  |  |  |  |  |  |  |  |  |  |  |  |  |  |  |  |  |  |  |  |  |  |  |  |  |  |  | 0% | 96% | 27 |
| 0.00 | when | CD66c | < | 1 |  |  | & | CD10 | >= |  |  | 36 | & | CD45 | < | 2 | & | CD24 | is | 38 | to | 58 | & | CD13 | < | 4 |  |  |  |  |  |  |  |  |  |  |  |  |  |  | & | CyIgM | >= | 5 |  |  |  |  |  |  |  |  |  |  |  |  |  |  |  |  |  |  |  |  | 0% | 96% | 28 |
| 0.00 | when | CD66c | < | 1 |  |  | & | CD10 | >= |  |  | 36 | & | CD45 | < | 2 | & | CD24 | is | 38 | to | 58 | & | CD13 | < | 4 |  |  |  |  |  |  |  |  |  |  |  |  |  |  | & | CyIgM | < | 5 |  |  |  |  | & | TdT | >= | 12 |  |  |  |  |  |  |  |  |  |  |  |  | 0% | 96% | 29 |
| 0.00 | when | CD66c | < | 1 |  |  | & | CD10 | >= |  |  | 95 | & | CD45 | < | 2 | & | CD24 | < | 26 |  |  |  |  |  |  |  |  |  |  |  |  |  |  |  |  | & | CD34 | < | 4 |  |  |  |  |  |  |  |  |  |  |  |  |  |  |  |  |  |  |  |  |  |  |  |  | 0% | 96% | 30 |
| 0.50 | when | CD66c | >= |  |  | 1 | & | CD10 | is | 25 | to | 98 | & | CD45 | >= | 4 | & | CD24 | < | 101 |  |  | & | CD13 | < | 24 | & | CD38 | < | 20 | & | CD22 | >= |  |  | 2 |  |  |  |  |  |  |  |  |  |  |  |  |  |  |  |  |  |  |  |  |  |  |  |  |  |  |  |  | 0% | 96% | 31 |
| 0.50 | when | CD66c | >= |  |  | 1 | & | CD10 | < | 98 |  |  |  |  |  |  | & | CD24 | >= |  |  | 101 | & | CD13 | < | 24 | & | CD38 | < | 20 |  |  |  |  |  |  | & | CD34 | < | 2 | & | CyIgM | >= | 5 |  |  |  |  |  |  |  |  |  |  |  |  |  |  |  |  |  |  |  |  | 0% | 96% | 32 |
| 0.50 | when | CD66c | < | 1 |  |  |  |  |  |  |  |  | & | CD45 | < | 2 | & | CD24 | is | 26 | to | 35 |  |  |  |  |  |  |  |  |  |  |  |  |  |  | & | CD34 | < | 4 | & | CyIgM | >= | 3 |  |  |  |  |  |  |  |  |  |  |  |  |  |  |  |  |  |  |  |  | 0% | 96% | 33 |
| 1.00 | when | CD66c | is | 1 | to | 4 | & | CD10 | is | 25 | to | 98 |  |  |  |  | & | CD24 | < | 101 |  |  | & | CD13 | < | 24 | & | CD38 | < | 20 | & | CD22 | < | 2 |  |  |  |  |  |  |  |  |  |  |  |  |  |  |  |  |  |  |  |  |  |  |  |  |  |  |  |  |  |  | 0% | 96% | 34 |
| 1.00 | when | CD66c | >= |  |  | 1 | & | CD10 | < | 98 |  |  |  |  |  |  | & | CD24 | >= |  |  | 101 | & | CD13 | < | 24 | & | CD38 | < | 20 |  |  |  |  |  |  | & | CD34 | >= | 2 | & | CyIgM | >= | 5 |  |  |  |  |  |  |  |  |  |  |  |  |  |  |  |  |  |  |  |  | 0% | 96% | 35 |
| 1.00 | when | CD66c | >= |  |  | 1 | & | CD10 | < | 98 |  |  |  |  |  |  |  |  |  |  |  |  | & | CD13 | < | 24 | & | CD38 | >= | 20 |  |  |  |  |  |  |  |  |  |  |  |  |  |  |  |  |  |  |  |  |  |  |  |  |  |  |  |  |  |  |  |  |  |  | 0% | 96% | 36 |
| 1.00 | when | CD66c | >= |  |  | 1 | & | CD10 | < | 98 |  |  |  |  |  |  |  |  |  |  |  |  | & | CD13 | >= | 24 |  |  |  |  |  |  |  |  |  |  |  |  |  |  |  |  |  |  |  |  |  |  |  |  |  |  |  |  |  |  |  |  |  |  |  |  |  |  | 0% | 96% | 37 |
| 1.00 | when | CD66c | >= |  |  | 1 | & | CD10 | >= |  |  | 98 |  |  |  |  |  |  |  |  |  |  |  |  |  |  |  |  |  |  |  |  |  |  |  |  |  |  |  |  |  |  |  |  | & | CD33 | >= | 1 |  |  |  |  |  |  |  |  |  |  |  |  |  |  |  |  | 0% | 96% | 38 |
| 1.00 | when | CD66c | < | 1 |  |  | & | CD10 | < | 16 |  |  | & | CD45 | >= | 2 |  |  |  |  |  |  |  |  |  |  |  |  |  |  | & | CD22 | < | 3 |  |  |  |  |  |  |  |  |  |  |  |  |  |  | & | TdT | >= | 5 | & | CD81 | < | 3 |  |  |  |  |  |  |  |  | 0% | 96% | 39 |
| 1.00 | when | CD66c | < | 1 |  |  | & | CD10 | >= |  |  | 17 | & | CD45 | >= | 2 |  |  |  |  |  |  |  |  |  |  |  |  |  |  | & | CD22 | < | 2 |  |  |  |  |  |  |  |  |  |  |  |  |  |  |  |  |  |  |  |  |  |  |  |  |  |  | & | CD123 | >= | 1 | 0% | 96% | 40 |
| 1.00 | when | CD66c | < | 1 |  |  | & | CD10 | is | 16 | to | 17 | & | CD45 | >= | 2 |  |  |  |  |  |  |  |  |  |  |  |  |  |  | & | CD22 | < | 2 |  |  |  |  |  |  |  |  |  |  |  |  |  |  |  |  |  |  |  |  |  |  |  |  |  |  |  |  |  |  | 0% | 96% | 41 |
| 1.00 | when | CD66c | < | 1 |  |  | & | CD10 | is | 16 | to | 50 | & | CD45 | >= | 2 |  |  |  |  |  |  |  |  |  |  |  |  |  |  | & | CD22 | is | 2 | to | 3 |  |  |  |  |  |  |  |  |  |  |  |  |  |  |  |  | & | CD81 | >= | 3 |  |  |  |  |  |  |  |  | 0% | 96% | 42 |
| 1.00 | when | CD66c | < | 1 |  |  | & | CD10 | >= |  |  | 71 | & | CD45 | >= | 2 |  |  |  |  |  |  |  |  |  |  |  |  |  |  | & | CD22 | is | 2 | to | 3 |  |  |  |  |  |  |  |  |  |  |  |  |  |  |  |  |  |  |  |  |  |  |  |  |  |  |  |  | 0% | 96% | 43 |
| 1.00 | when | CD66c | < | 1 |  |  | & | CD10 | < | 54 |  |  | & | CD45 | < | 2 | & | CD24 | < | 38 |  |  |  |  |  |  | & | CD38 | >= | 2 |  |  |  |  |  |  | & | CD34 | >= | 4 |  |  |  |  | & | CD33 | >= | 1 |  |  |  |  |  |  |  |  |  |  |  |  |  |  |  |  | 0% | 96% | 44 |

#### t(v;11q23)/KMT2A

```
Confusion Matrix and Statistics

          Reference
Prediction   0   1
         0 588   5
         1   0  12
                                          
               Accuracy : 0.9917          
                 95% CI : (0.9808, 0.9973)
    No Information Rate : 0.9719          
    P-Value [Acc > NIR] : 0.0005952       
                                          
                  Kappa : 0.8235          
                                          
 Mcnemar's Test P-Value : 0.0736383       
                                          
            Sensitivity : 0.70588         
            Specificity : 1.00000         
         Pos Pred Value : 1.00000         
         Neg Pred Value : 0.99157         
              Precision : 1.00000         
                 Recall : 0.70588         
                     F1 : 0.82759         
             Prevalence : 0.02810         
         Detection Rate : 0.01983         
   Detection Prevalence : 0.01983         
      Balanced Accuracy : 0.85294         
                                          
       'Positive' Class : 1
```

|  | Variable importance |
| --- | --- |
| NG2 | 12.7604797 |
| CD24 | 3.0000000 |
| CyIgM | 2.7025134 |
| CD34 | 2.2500000 |
| CD123 | 1.9693179 |
| CD10 | 1.6666667 |
| CD45 | 1.5000000 |
| CD38 | 0.7500000 |
| CD20 | 0.2962410 |
| CD15\_CD65 | 0.2598018 |

| probability: primary class | x | x\_2 | x\_3 | x\_4 | x\_5 | x\_6 | x\_7 | x\_8 | x\_9 | x\_10 | x\_11 | x\_12 | x\_13 | x\_14 | x\_15 | x\_16 | x\_17 | x\_18 | x\_19 | x\_20 | x\_21 | x\_22 | x\_23 | x\_24 | x\_25 | x\_26 | x\_27 | x\_28 | x\_29 | x\_30 | x\_31 | x\_32 | cover | cover\_cumulative | rule\_no |
| --- | --- | --- | --- | --- | --- | --- | --- | --- | --- | --- | --- | --- | --- | --- | --- | --- | --- | --- | --- | --- | --- | --- | --- | --- | --- | --- | --- | --- | --- | --- | --- | --- | --- | --- | --- |
| 0.01 | when | NG2 | is | 0 | & | CD123 | < | 9 | & | CyIgM | < | 18 | & | CD20 | < | 9 |  |  |  |  |  |  |  |  | & | CD15\_CD65 | < | 1 |  |  |  |  | 96% | 96% | 1 |
| 0.00 | when | NG2 | is | 0 | & | CD123 | < | 9 | & | CyIgM | < | 18 | & | CD20 | >= | 9 |  |  |  |  | & | CD10 | >= | 26 |  |  |  |  |  |  |  |  | 1% | 97% | 2 |
| 0.14 | when | NG2 | is | 0 | & | CD123 | < | 9 | & | CyIgM | < | 18 | & | CD20 | < | 9 |  |  |  |  |  |  |  |  | & | CD15\_CD65 | >= | 1 |  |  |  |  | 1% | 98% | 3 |
| 1.00 | when | NG2 | is | 1 |  |  |  |  |  |  |  |  |  |  |  |  | & | CD24 | < | 5 |  |  |  |  |  |  |  |  |  |  |  |  | 1% | 99% | 4 |
| 0.00 | when | NG2 | is | 1 |  |  |  |  |  |  |  |  |  |  |  |  | & | CD24 | >= | 5 |  |  |  |  |  |  |  |  | & | CD34 | < | 16 | 0% | 99% | 5 |
| 1.00 | when | NG2 | is | 0 | & | CD123 | < | 9 | & | CyIgM | < | 18 | & | CD20 | >= | 9 |  |  |  |  | & | CD10 | < | 26 |  |  |  |  |  |  |  |  | 0% | 99% | 6 |
| 1.00 | when | NG2 | is | 0 | & | CD123 | < | 9 | & | CyIgM | >= | 18 |  |  |  |  |  |  |  |  |  |  |  |  |  |  |  |  |  |  |  |  | 0% | 99% | 7 |
| 1.00 | when | NG2 | is | 0 | & | CD123 | >= | 9 |  |  |  |  |  |  |  |  |  |  |  |  |  |  |  |  |  |  |  |  |  |  |  |  | 0% | 99% | 8 |
| 1.00 | when | NG2 | is | 1 |  |  |  |  |  |  |  |  |  |  |  |  | & | CD24 | >= | 5 |  |  |  |  |  |  |  |  | & | CD34 | >= | 16 | 0% | 99% | 9 |

#### hyperdiploidy

```
Confusion Matrix and Statistics

          Reference
Prediction   0   1
         0 361  17
         1  17 107
                                          
               Accuracy : 0.9323          
                 95% CI : (0.9066, 0.9526)
    No Information Rate : 0.753           
    P-Value [Acc > NIR] : <2e-16          
                                          
                  Kappa : 0.8179          
                                          
 Mcnemar's Test P-Value : 1               
                                          
            Sensitivity : 0.8629          
            Specificity : 0.9550          
         Pos Pred Value : 0.8629          
         Neg Pred Value : 0.9550          
              Precision : 0.8629          
                 Recall : 0.8629          
                     F1 : 0.8629          
             Prevalence : 0.2470          
         Detection Rate : 0.2131          
   Detection Prevalence : 0.2470          
      Balanced Accuracy : 0.9090          
                                          
       'Positive' Class : 1
```

|  | Variable importance |
| --- | --- |
| CD123 | 35.622886 |
| CD24 | 32.714526 |
| CD10 | 25.145816 |
| CD34 | 16.439912 |
| CD9 | 14.285679 |
| TdT | 12.901010 |
| CD45 | 10.271138 |
| CD13 | 8.445808 |
| CD22 | 6.269507 |
| CyIgM | 5.945709 |
| CD38 | 5.340533 |
| CD66c | 5.172637 |
| CD81 | 4.062801 |
| CD20 | 3.455975 |
| CD33 | 2.669710 |

| probability: primary class | x | x\_2 | x\_3 | x\_4 | x\_5 | x\_6 | x\_7 | x\_8 | x\_9 | x\_10 | x\_11 | x\_12 | x\_13 | x\_14 | x\_15 | x\_16 | x\_17 | x\_18 | x\_19 | x\_20 | x\_21 | x\_22 | x\_23 | x\_24 | x\_25 | x\_26 | x\_27 | x\_28 | x\_29 | x\_30 | x\_31 | x\_32 | x\_33 | x\_34 | x\_35 | x\_36 | x\_37 | x\_38 | x\_39 | x\_40 | x\_41 | x\_42 | x\_43 | x\_44 | x\_45 | x\_46 | x\_47 | x\_48 | x\_49 | x\_50 | x\_51 | x\_52 | x\_53 | x\_54 | x\_55 | x\_56 | x\_57 | x\_58 | x\_59 | x\_60 | x\_61 | x\_62 | x\_63 | x\_64 | x\_65 | x\_66 | x\_67 | x\_68 | x\_69 | x\_70 | x\_71 | x\_72 | x\_73 | x\_74 | cover | cover\_cumulative | rule\_no |
| --- | --- | --- | --- | --- | --- | --- | --- | --- | --- | --- | --- | --- | --- | --- | --- | --- | --- | --- | --- | --- | --- | --- | --- | --- | --- | --- | --- | --- | --- | --- | --- | --- | --- | --- | --- | --- | --- | --- | --- | --- | --- | --- | --- | --- | --- | --- | --- | --- | --- | --- | --- | --- | --- | --- | --- | --- | --- | --- | --- | --- | --- | --- | --- | --- | --- | --- | --- | --- | --- | --- | --- | --- | --- | --- | --- | --- | --- |
| 0.03 | when | CD123 | < | 1 |  |  | & | CD24 | < | 124 |  |  | & | CD9 | < | 22 |  |  | & | CD10 | is | 3 | to | 76 | & | CD13 | < | 16 | & | TdT | < | 14 |  |  | & | CD81 | < | 13 |  |  | & | CD66c | >= | 1 |  |  |  |  |  |  |  |  |  |  |  |  |  |  | & | CD45 | < | 4 |  |  |  |  |  |  |  |  |  |  |  |  | 14% | 14% | 1 |
| 0.15 | when | CD123 | < | 1 |  |  | & | CD24 | < | 39 |  |  |  |  |  |  |  |  | & | CD10 | is | 6 | to | 46 | & | CD13 | < | 16 | & | TdT | < | 9 |  |  | & | CD81 | < | 13 |  |  | & | CD66c | < | 1 |  |  |  |  |  |  |  |  |  |  |  |  |  |  |  |  |  |  |  |  |  |  |  |  |  |  |  |  |  |  | 12% | 26% | 2 |
| 0.00 | when | CD123 | < | 1 |  |  | & | CD24 | >= |  |  | 40 |  |  |  |  |  |  |  |  |  |  |  |  | & | CD13 | < | 16 | & | TdT | < | 9 |  |  | & | CD81 | < | 13 |  |  | & | CD66c | < | 1 |  |  |  |  |  |  |  |  |  |  |  |  |  |  |  |  |  |  |  |  |  |  |  |  |  |  |  |  |  |  | 9% | 35% | 3 |
| 0.00 | when | CD123 | < | 1 |  |  | & | CD24 | < | 39 |  |  |  |  |  |  |  |  | & | CD10 | >= |  |  | 47 | & | CD13 | < | 16 | & | TdT | < | 9 |  |  | & | CD81 | < | 13 |  |  | & | CD66c | < | 1 |  |  |  |  |  |  |  |  |  |  |  |  |  |  |  |  |  |  |  |  |  |  |  |  |  |  |  |  |  |  | 8% | 43% | 4 |
| 0.00 | when | CD123 | < | 1 |  |  | & | CD24 | < | 39 |  |  |  |  |  |  |  |  | & | CD10 | < | 6 |  |  | & | CD13 | < | 16 | & | TdT | < | 9 |  |  | & | CD81 | < | 13 |  |  | & | CD66c | < | 1 |  |  |  |  |  |  |  |  |  |  |  |  |  |  |  |  |  |  |  |  |  |  |  |  |  |  |  |  |  |  | 7% | 50% | 5 |
| 0.81 | when | CD123 | >= |  |  | 1 | & | CD24 | is | 15 | to | 61 | & | CD9 | >= |  |  | 3 | & | CD10 | < | 32 |  |  |  |  |  |  |  |  |  |  |  |  |  |  |  |  |  |  |  |  |  |  | & | CD38 | < | 6 | & | CD34 | < | 12 |  |  |  |  |  |  |  |  |  |  | & | CD33 | < | 1 | & | CD20 | < | 2 |  |  |  |  | 7% | 57% | 6 |
| 0.00 | when | CD123 | is | 1 | to | 2 |  |  |  |  |  |  | & | CD9 | < | 3 |  |  |  |  |  |  |  |  |  |  |  |  |  |  |  |  |  |  |  |  |  |  |  |  |  |  |  |  |  |  |  |  |  |  |  |  |  |  |  |  |  |  |  |  |  |  |  |  |  |  |  |  |  |  |  |  |  |  | 5% | 62% | 7 |
| 1.00 | when | CD123 | >= |  |  | 1 | & | CD24 | is | 14 | to | 61 | & | CD9 | >= |  |  | 3 | & | CD10 | >= |  |  | 8 |  |  |  |  |  |  |  |  |  |  |  |  |  |  |  |  |  |  |  |  |  |  |  |  | & | CD34 | >= | 12 |  |  |  |  |  |  |  |  |  |  | & | CD33 | < | 1 | & | CD20 | < | 2 |  |  |  |  | 4% | 66% | 8 |
| 0.00 | when | CD123 | >= |  |  | 1 | & | CD24 | < | 14 |  |  | & | CD9 | >= |  |  | 3 |  |  |  |  |  |  |  |  |  |  | & | TdT | < | 7 |  |  |  |  |  |  |  |  |  |  |  |  | & | CD38 | < | 5 |  |  |  |  | & | CD22 | >= |  |  | 2 | & | CD45 | >= | 2 |  |  |  |  |  |  |  |  |  |  |  |  | 3% | 69% | 9 |
| 0.08 | when | CD123 | >= |  |  | 1 | & | CD24 | is | 8 | to | 14 | & | CD9 | is | 3 | to | 13 |  |  |  |  |  |  |  |  |  |  | & | TdT | < | 7 |  |  |  |  |  |  |  |  |  |  |  |  | & | CD38 | < | 5 | & | CD34 | >= | 2 | & | CD22 | >= |  |  | 2 | & | CD45 | < | 2 |  |  |  |  |  |  |  |  |  |  |  |  | 3% | 72% | 10 |
| 0.62 | when | CD123 | >= |  |  | 1 | & | CD24 | is | 14 | to | 61 | & | CD9 | >= |  |  | 3 | & | CD10 | >= |  |  | 32 |  |  |  |  |  |  |  |  |  |  |  |  |  |  |  |  |  |  |  |  | & | CD38 | < | 6 | & | CD34 | < | 12 |  |  |  |  |  |  |  |  |  |  | & | CD33 | < | 1 | & | CD20 | < | 2 | & | CyIgM | < | 3 | 3% | 75% | 11 |
| 0.00 | when | CD123 | < | 1 |  |  | & | CD24 | < | 124 |  |  | & | CD9 | < | 22 |  |  | & | CD10 | < | 3 |  |  | & | CD13 | < | 16 |  |  |  |  |  |  | & | CD81 | is | 3 | to | 13 | & | CD66c | >= | 1 |  |  |  |  |  |  |  |  | & | CD22 | >= |  |  | 2 |  |  |  |  |  |  |  |  |  |  |  |  |  |  |  |  | 2% | 77% | 12 |
| 0.00 | when | CD123 | >= |  |  | 1 | & | CD24 | is | 61 | to | 126 | & | CD9 | >= |  |  | 5 |  |  |  |  |  |  |  |  |  |  |  |  |  |  |  |  |  |  |  |  |  |  |  |  |  |  |  |  |  |  |  |  |  |  |  |  |  |  |  |  |  |  |  |  |  |  |  |  |  |  |  |  |  |  |  |  | 2% | 79% | 13 |
| 0.00 | when | CD123 | < | 1 |  |  | & | CD24 | >= |  |  | 15 |  |  |  |  |  |  | & | CD10 | >= |  |  | 2 | & | CD13 | < | 16 | & | TdT | >= |  |  | 9 | & | CD81 | < | 13 |  |  | & | CD66c | < | 1 |  |  |  |  |  |  |  |  | & | CD22 | >= |  |  | 2 |  |  |  |  |  |  |  |  |  |  |  |  |  |  |  |  | 1% | 80% | 14 |
| 0.00 | when | CD123 | < | 1 |  |  |  |  |  |  |  |  |  |  |  |  |  |  | & | CD10 | >= |  |  | 80 | & | CD13 | < | 16 |  |  |  |  |  |  | & | CD81 | < | 13 |  |  | & | CD66c | >= | 1 |  |  |  |  | & | CD34 | < | 2 |  |  |  |  |  |  |  |  |  |  |  |  |  |  |  |  |  |  |  |  |  |  | 1% | 81% | 15 |
| 0.00 | when | CD123 | < | 1 |  |  |  |  |  |  |  |  |  |  |  |  |  |  |  |  |  |  |  |  | & | CD13 | < | 16 | & | TdT | < | 7 |  |  | & | CD81 | >= |  |  | 13 |  |  |  |  |  |  |  |  |  |  |  |  |  |  |  |  |  |  |  |  |  |  |  |  |  |  |  |  |  |  |  |  |  |  | 1% | 82% | 16 |
| 0.00 | when | CD123 | >= |  |  | 2 |  |  |  |  |  |  | & | CD9 | < | 3 |  |  |  |  |  |  |  |  |  |  |  |  |  |  |  |  |  |  |  |  |  |  |  |  |  |  |  |  |  |  |  |  |  |  |  |  |  |  |  |  |  |  | & | CD45 | >= | 3 |  |  |  |  |  |  |  |  |  |  |  |  | 1% | 83% | 17 |
| 0.00 | when | CD123 | >= |  |  | 2 | & | CD24 | < | 8 |  |  | & | CD9 | >= |  |  | 3 |  |  |  |  |  |  |  |  |  |  | & | TdT | is | 4 | to | 7 |  |  |  |  |  |  |  |  |  |  | & | CD38 | < | 5 |  |  |  |  | & | CD22 | >= |  |  | 2 | & | CD45 | < | 2 |  |  |  |  |  |  |  |  |  |  |  |  | 1% | 84% | 18 |
| 0.00 | when | CD123 | >= |  |  | 1 | & | CD24 | is | 14 | to | 61 | & | CD9 | >= |  |  | 3 | & | CD10 | < | 71 |  |  |  |  |  |  |  |  |  |  |  |  |  |  |  |  |  |  |  |  |  |  |  |  |  |  |  |  |  |  |  |  |  |  |  |  |  |  |  |  | & | CD33 | >= | 1 |  |  |  |  |  |  |  |  | 1% | 85% | 19 |
| 0.20 | when | CD123 | >= |  |  | 1 | & | CD24 | is | 14 | to | 61 | & | CD9 | >= |  |  | 3 | & | CD10 | >= |  |  | 32 |  |  |  |  |  |  |  |  |  |  |  |  |  |  |  |  |  |  |  |  | & | CD38 | < | 6 | & | CD34 | < | 12 |  |  |  |  |  |  |  |  |  |  | & | CD33 | < | 1 | & | CD20 | < | 2 | & | CyIgM | >= | 3 | 1% | 86% | 20 |
| 0.25 | when | CD123 | < | 1 |  |  | & | CD24 | >= |  |  | 15 |  |  |  |  |  |  | & | CD10 | >= |  |  | 2 | & | CD13 | < | 16 | & | TdT | >= |  |  | 9 | & | CD81 | < | 13 |  |  | & | CD66c | < | 1 |  |  |  |  |  |  |  |  | & | CD22 | < | 2 |  |  |  |  |  |  |  |  |  |  |  |  |  |  |  |  |  |  | 1% | 87% | 21 |
| 0.25 | when | CD123 | < | 1 |  |  | & | CD24 | < | 124 |  |  | & | CD9 | < | 22 |  |  | & | CD10 | is | 3 | to | 76 | & | CD13 | < | 16 | & | TdT | < | 14 |  |  | & | CD81 | < | 13 |  |  | & | CD66c | >= | 1 |  |  |  |  |  |  |  |  |  |  |  |  |  |  | & | CD45 | >= | 4 |  |  |  |  |  |  |  |  |  |  |  |  | 1% | 88% | 22 |
| 0.67 | when | CD123 | < | 1 |  |  | & | CD24 | < | 124 |  |  | & | CD9 | < | 22 |  |  | & | CD10 | < | 3 |  |  | & | CD13 | < | 16 |  |  |  |  |  |  | & | CD81 | < | 3 |  |  | & | CD66c | >= | 1 |  |  |  |  |  |  |  |  |  |  |  |  |  |  |  |  |  |  |  |  |  |  |  |  |  |  |  |  |  |  | 1% | 89% | 23 |
| 0.67 | when | CD123 | < | 1 |  |  | & | CD24 | >= |  |  | 124 |  |  |  |  |  |  | & | CD10 | < | 76 |  |  | & | CD13 | < | 16 |  |  |  |  |  |  | & | CD81 | < | 13 |  |  | & | CD66c | >= | 1 |  |  |  |  |  |  |  |  |  |  |  |  |  |  |  |  |  |  |  |  |  |  |  |  |  |  |  |  |  |  | 1% | 90% | 24 |
| 0.67 | when | CD123 | >= |  |  | 1 | & | CD24 | is | 14 | to | 61 | & | CD9 | >= |  |  | 3 | & | CD10 | is | 3 | to | 8 |  |  |  |  |  |  |  |  |  |  |  |  |  |  |  |  |  |  |  |  |  |  |  |  | & | CD34 | >= | 12 |  |  |  |  |  |  |  |  |  |  | & | CD33 | < | 1 | & | CD20 | < | 2 |  |  |  |  | 1% | 91% | 25 |
| 0.75 | when | CD123 | < | 1 |  |  | & | CD24 | is | 39 | to | 40 |  |  |  |  |  |  |  |  |  |  |  |  | & | CD13 | < | 16 | & | TdT | < | 9 |  |  | & | CD81 | < | 13 |  |  | & | CD66c | < | 1 |  |  |  |  |  |  |  |  |  |  |  |  |  |  |  |  |  |  |  |  |  |  |  |  |  |  |  |  |  |  | 1% | 92% | 26 |
| 1.00 | when | CD123 | < | 1 |  |  |  |  |  |  |  |  |  |  |  |  |  |  | & | CD10 | >= |  |  | 76 | & | CD13 | < | 16 |  |  |  |  |  |  | & | CD81 | < | 13 |  |  | & | CD66c | >= | 1 |  |  |  |  | & | CD34 | >= | 2 |  |  |  |  |  |  |  |  |  |  |  |  |  |  |  |  |  |  |  |  |  |  | 1% | 93% | 27 |
| 1.00 | when | CD123 | < | 1 |  |  |  |  |  |  |  |  |  |  |  |  |  |  | & | CD10 | >= |  |  | 14 | & | CD13 | >= | 16 |  |  |  |  |  |  |  |  |  |  |  |  |  |  |  |  |  |  |  |  |  |  |  |  |  |  |  |  |  |  |  |  |  |  |  |  |  |  |  |  |  |  |  |  |  |  | 1% | 94% | 28 |
| 1.00 | when | CD123 | >= |  |  | 2 |  |  |  |  |  |  | & | CD9 | < | 3 |  |  |  |  |  |  |  |  | & | CD13 | < | 3 |  |  |  |  |  |  |  |  |  |  |  |  |  |  |  |  |  |  |  |  |  |  |  |  |  |  |  |  |  |  | & | CD45 | < | 3 |  |  |  |  |  |  |  |  |  |  |  |  | 1% | 95% | 29 |
| 1.00 | when | CD123 | is | 1 | to | 2 | & | CD24 | < | 8 |  |  | & | CD9 | >= |  |  | 3 |  |  |  |  |  |  |  |  |  |  | & | TdT | < | 7 |  |  |  |  |  |  |  |  |  |  |  |  | & | CD38 | < | 5 |  |  |  |  | & | CD22 | >= |  |  | 2 | & | CD45 | < | 2 |  |  |  |  |  |  |  |  |  |  |  |  | 1% | 96% | 30 |
| 1.00 | when | CD123 | >= |  |  | 1 | & | CD24 | < | 14 |  |  | & | CD9 | >= |  |  | 3 |  |  |  |  |  |  |  |  |  |  | & | TdT | >= |  |  | 7 |  |  |  |  |  |  |  |  |  |  | & | CD38 | < | 5 | & | CD34 | < | 21 | & | CD22 | is | 2 | to | 5 | & | CD45 | < | 3 |  |  |  |  |  |  |  |  |  |  |  |  | 1% | 97% | 31 |
| 0.00 | when | CD123 | < | 1 |  |  |  |  |  |  |  |  |  |  |  |  |  |  | & | CD10 | < | 14 |  |  | & | CD13 | >= | 16 |  |  |  |  |  |  |  |  |  |  |  |  |  |  |  |  |  |  |  |  |  |  |  |  |  |  |  |  |  |  |  |  |  |  |  |  |  |  |  |  |  |  |  |  |  |  | 0% | 97% | 32 |
| 0.00 | when | CD123 | >= |  |  | 2 |  |  |  |  |  |  | & | CD9 | < | 3 |  |  |  |  |  |  |  |  | & | CD13 | >= | 3 |  |  |  |  |  |  |  |  |  |  |  |  |  |  |  |  |  |  |  |  |  |  |  |  |  |  |  |  |  |  | & | CD45 | < | 3 |  |  |  |  |  |  |  |  |  |  |  |  | 0% | 97% | 33 |
| 0.00 | when | CD123 | >= |  |  | 1 | & | CD24 | < | 14 |  |  | & | CD9 | >= |  |  | 3 |  |  |  |  |  |  |  |  |  |  | & | TdT | >= |  |  | 7 |  |  |  |  |  |  |  |  |  |  | & | CD38 | < | 5 |  |  |  |  | & | CD22 | >= |  |  | 5 |  |  |  |  |  |  |  |  |  |  |  |  |  |  |  |  | 0% | 97% | 34 |
| 0.00 | when | CD123 | >= |  |  | 1 | & | CD24 | < | 14 |  |  | & | CD9 | >= |  |  | 3 |  |  |  |  |  |  |  |  |  |  | & | TdT | >= |  |  | 7 |  |  |  |  |  |  |  |  |  |  | & | CD38 | < | 5 | & | CD34 | >= | 21 | & | CD22 | is | 2 | to | 5 |  |  |  |  |  |  |  |  |  |  |  |  |  |  |  |  | 0% | 97% | 35 |
| 0.00 | when | CD123 | >= |  |  | 1 | & | CD24 | < | 14 |  |  | & | CD9 | >= |  |  | 3 |  |  |  |  |  |  |  |  |  |  | & | TdT | >= |  |  | 7 |  |  |  |  |  |  |  |  |  |  | & | CD38 | < | 5 | & | CD34 | < | 21 | & | CD22 | is | 2 | to | 5 | & | CD45 | >= | 3 |  |  |  |  |  |  |  |  |  |  |  |  | 0% | 97% | 36 |
| 0.00 | when | CD123 | >= |  |  | 1 | & | CD24 | is | 14 | to | 61 | & | CD9 | >= |  |  | 3 |  |  |  |  |  |  |  |  |  |  |  |  |  |  |  |  |  |  |  |  |  |  |  |  |  |  |  |  |  |  |  |  |  |  |  |  |  |  |  |  |  |  |  |  | & | CD33 | < | 1 | & | CD20 | >= | 2 |  |  |  |  | 0% | 97% | 37 |
| 0.00 | when | CD123 | >= |  |  | 1 | & | CD24 | is | 14 | to | 61 | & | CD9 | >= |  |  | 3 |  |  |  |  |  |  |  |  |  |  |  |  |  |  |  |  |  |  |  |  |  |  |  |  |  |  | & | CD38 | >= | 6 | & | CD34 | < | 12 |  |  |  |  |  |  |  |  |  |  | & | CD33 | < | 1 | & | CD20 | < | 2 |  |  |  |  | 0% | 97% | 38 |
| 0.00 | when | CD123 | >= |  |  | 1 | & | CD24 | is | 14 | to | 15 | & | CD9 | >= |  |  | 3 | & | CD10 | < | 32 |  |  |  |  |  |  |  |  |  |  |  |  |  |  |  |  |  |  |  |  |  |  | & | CD38 | < | 6 | & | CD34 | < | 12 |  |  |  |  |  |  |  |  |  |  | & | CD33 | < | 1 | & | CD20 | < | 2 |  |  |  |  | 0% | 97% | 39 |
| 0.00 | when | CD123 | >= |  |  | 1 | & | CD24 | is | 14 | to | 61 | & | CD9 | >= |  |  | 3 | & | CD10 | < | 3 |  |  |  |  |  |  |  |  |  |  |  |  |  |  |  |  |  |  |  |  |  |  |  |  |  |  | & | CD34 | >= | 12 |  |  |  |  |  |  |  |  |  |  | & | CD33 | < | 1 | & | CD20 | < | 2 |  |  |  |  | 0% | 97% | 40 |
| 0.50 | when | CD123 | < | 1 |  |  | & | CD24 | < | 124 |  |  | & | CD9 | < | 22 |  |  | & | CD10 | is | 3 | to | 76 | & | CD13 | < | 16 | & | TdT | >= |  |  | 14 | & | CD81 | < | 13 |  |  | & | CD66c | >= | 1 |  |  |  |  |  |  |  |  |  |  |  |  |  |  |  |  |  |  |  |  |  |  |  |  |  |  |  |  |  |  | 0% | 97% | 41 |
| 0.50 | when | CD123 | >= |  |  | 1 | & | CD24 | is | 8 | to | 14 | & | CD9 | is | 3 | to | 13 |  |  |  |  |  |  |  |  |  |  | & | TdT | < | 7 |  |  |  |  |  |  |  |  |  |  |  |  | & | CD38 | < | 5 | & | CD34 | < | 2 | & | CD22 | >= |  |  | 2 | & | CD45 | < | 2 |  |  |  |  |  |  |  |  |  |  |  |  | 0% | 97% | 42 |
| 1.00 | when | CD123 | < | 1 |  |  | & | CD24 | < | 39 |  |  |  |  |  |  |  |  | & | CD10 | is | 46 | to | 47 | & | CD13 | < | 16 | & | TdT | < | 9 |  |  | & | CD81 | < | 13 |  |  | & | CD66c | < | 1 |  |  |  |  |  |  |  |  |  |  |  |  |  |  |  |  |  |  |  |  |  |  |  |  |  |  |  |  |  |  | 0% | 97% | 43 |
| 1.00 | when | CD123 | < | 1 |  |  | & | CD24 | >= |  |  | 15 |  |  |  |  |  |  | & | CD10 | < | 2 |  |  | & | CD13 | < | 16 | & | TdT | >= |  |  | 9 | & | CD81 | < | 13 |  |  | & | CD66c | < | 1 |  |  |  |  |  |  |  |  |  |  |  |  |  |  |  |  |  |  |  |  |  |  |  |  |  |  |  |  |  |  | 0% | 97% | 44 |
| 1.00 | when | CD123 | < | 1 |  |  | & | CD24 | < | 15 |  |  |  |  |  |  |  |  |  |  |  |  |  |  | & | CD13 | < | 16 | & | TdT | >= |  |  | 9 | & | CD81 | < | 13 |  |  | & | CD66c | < | 1 |  |  |  |  |  |  |  |  |  |  |  |  |  |  |  |  |  |  |  |  |  |  |  |  |  |  |  |  |  |  | 0% | 97% | 45 |
| 1.00 | when | CD123 | < | 1 |  |  | & | CD24 | < | 124 |  |  | & | CD9 | < | 22 |  |  | & | CD10 | < | 3 |  |  | & | CD13 | < | 16 |  |  |  |  |  |  | & | CD81 | is | 3 | to | 13 | & | CD66c | >= | 1 |  |  |  |  |  |  |  |  | & | CD22 | < | 2 |  |  |  |  |  |  |  |  |  |  |  |  |  |  |  |  |  |  | 0% | 97% | 46 |
| 1.00 | when | CD123 | < | 1 |  |  | & | CD24 | < | 124 |  |  | & | CD9 | >= |  |  | 22 | & | CD10 | < | 76 |  |  | & | CD13 | < | 16 |  |  |  |  |  |  | & | CD81 | < | 13 |  |  | & | CD66c | >= | 1 |  |  |  |  |  |  |  |  |  |  |  |  |  |  |  |  |  |  |  |  |  |  |  |  |  |  |  |  |  |  | 0% | 97% | 47 |
| 1.00 | when | CD123 | < | 1 |  |  |  |  |  |  |  |  |  |  |  |  |  |  | & | CD10 | is | 76 | to | 80 | & | CD13 | < | 16 |  |  |  |  |  |  | & | CD81 | < | 13 |  |  | & | CD66c | >= | 1 |  |  |  |  | & | CD34 | < | 2 |  |  |  |  |  |  |  |  |  |  |  |  |  |  |  |  |  |  |  |  |  |  | 0% | 97% | 48 |
| 1.00 | when | CD123 | < | 1 |  |  |  |  |  |  |  |  |  |  |  |  |  |  |  |  |  |  |  |  | & | CD13 | < | 16 | & | TdT | >= |  |  | 7 | & | CD81 | >= |  |  | 13 |  |  |  |  |  |  |  |  |  |  |  |  |  |  |  |  |  |  |  |  |  |  |  |  |  |  |  |  |  |  |  |  |  |  | 0% | 97% | 49 |
| 1.00 | when | CD123 | >= |  |  | 1 | & | CD24 | is | 8 | to | 14 | & | CD9 | >= |  |  | 13 |  |  |  |  |  |  |  |  |  |  | & | TdT | < | 7 |  |  |  |  |  |  |  |  |  |  |  |  | & | CD38 | < | 5 |  |  |  |  | & | CD22 | >= |  |  | 2 | & | CD45 | < | 2 |  |  |  |  |  |  |  |  |  |  |  |  | 0% | 97% | 50 |
| 1.00 | when | CD123 | >= |  |  | 2 | & | CD24 | < | 8 |  |  | & | CD9 | >= |  |  | 3 |  |  |  |  |  |  |  |  |  |  | & | TdT | < | 4 |  |  |  |  |  |  |  |  |  |  |  |  | & | CD38 | < | 5 |  |  |  |  | & | CD22 | >= |  |  | 2 | & | CD45 | < | 2 |  |  |  |  |  |  |  |  |  |  |  |  | 0% | 97% | 51 |
| 1.00 | when | CD123 | >= |  |  | 1 | & | CD24 | < | 14 |  |  | & | CD9 | >= |  |  | 3 |  |  |  |  |  |  |  |  |  |  |  |  |  |  |  |  |  |  |  |  |  |  |  |  |  |  | & | CD38 | < | 5 |  |  |  |  | & | CD22 | < | 2 |  |  |  |  |  |  |  |  |  |  |  |  |  |  |  |  |  |  | 0% | 97% | 52 |
| 1.00 | when | CD123 | >= |  |  | 1 | & | CD24 | < | 14 |  |  | & | CD9 | >= |  |  | 3 |  |  |  |  |  |  |  |  |  |  |  |  |  |  |  |  |  |  |  |  |  |  |  |  |  |  | & | CD38 | >= | 5 |  |  |  |  |  |  |  |  |  |  |  |  |  |  |  |  |  |  |  |  |  |  |  |  |  |  | 0% | 97% | 53 |
| 1.00 | when | CD123 | >= |  |  | 1 | & | CD24 | >= |  |  | 126 | & | CD9 | >= |  |  | 5 |  |  |  |  |  |  |  |  |  |  |  |  |  |  |  |  |  |  |  |  |  |  |  |  |  |  |  |  |  |  |  |  |  |  |  |  |  |  |  |  |  |  |  |  |  |  |  |  |  |  |  |  |  |  |  |  | 0% | 97% | 54 |
| 1.00 | when | CD123 | >= |  |  | 1 | & | CD24 | >= |  |  | 61 | & | CD9 | is | 3 | to | 5 |  |  |  |  |  |  |  |  |  |  |  |  |  |  |  |  |  |  |  |  |  |  |  |  |  |  |  |  |  |  |  |  |  |  |  |  |  |  |  |  |  |  |  |  |  |  |  |  |  |  |  |  |  |  |  |  | 0% | 97% | 55 |
| 1.00 | when | CD123 | >= |  |  | 1 | & | CD24 | is | 14 | to | 61 | & | CD9 | >= |  |  | 3 | & | CD10 | >= |  |  | 71 |  |  |  |  |  |  |  |  |  |  |  |  |  |  |  |  |  |  |  |  |  |  |  |  |  |  |  |  |  |  |  |  |  |  |  |  |  |  | & | CD33 | >= | 1 |  |  |  |  |  |  |  |  | 0% | 97% | 56 |

#### no aberration (examples without any aberration)

```
Confusion Matrix and Statistics

          Reference
Prediction   0   1
         0 169  22
         1  21 235
                                          
               Accuracy : 0.9038          
                 95% CI : (0.8726, 0.9295)
    No Information Rate : 0.5749          
    P-Value [Acc > NIR] : <2e-16          
                                          
                  Kappa : 0.8033          
                                          
 Mcnemar's Test P-Value : 1               
                                          
            Sensitivity : 0.9144          
            Specificity : 0.8895          
         Pos Pred Value : 0.9180          
         Neg Pred Value : 0.8848          
              Precision : 0.9180          
                 Recall : 0.9144          
                     F1 : 0.9162          
             Prevalence : 0.5749          
         Detection Rate : 0.5257          
   Detection Prevalence : 0.5727          
      Balanced Accuracy : 0.9019          
                                          
       'Positive' Class : 1
```

|  | Variable importance |
| --- | --- |
| CD10 | 42.672515 |
| CD24 | 31.537127 |
| CD34 | 27.035310 |
| TdT | 24.513295 |
| CD123 | 22.748730 |
| CD66c | 14.527856 |
| CD45 | 12.209144 |
| CD22 | 11.395381 |
| CD81 | 9.367473 |
| CD9 | 6.341925 |
| CD38 | 6.141793 |
| CD13 | 5.609190 |
| NG2 | 4.523077 |
| CD20 | 3.539795 |
| CyIgM | 3.491281 |
| CD33 | 1.888889 |

| probability: primary class | x | x\_2 | x\_3 | x\_4 | x\_5 | x\_6 | x\_7 | x\_8 | x\_9 | x\_10 | x\_11 | x\_12 | x\_13 | x\_14 | x\_15 | x\_16 | x\_17 | x\_18 | x\_19 | x\_20 | x\_21 | x\_22 | x\_23 | x\_24 | x\_25 | x\_26 | x\_27 | x\_28 | x\_29 | x\_30 | x\_31 | x\_32 | x\_33 | x\_34 | x\_35 | x\_36 | x\_37 | x\_38 | x\_39 | x\_40 | x\_41 | x\_42 | x\_43 | x\_44 | x\_45 | x\_46 | x\_47 | x\_48 | x\_49 | x\_50 | x\_51 | x\_52 | x\_53 | x\_54 | x\_55 | x\_56 | x\_57 | x\_58 | x\_59 | x\_60 | x\_61 | x\_62 | x\_63 | x\_64 | x\_65 | x\_66 | x\_67 | x\_68 | x\_69 | x\_70 | x\_71 | x\_72 | x\_73 | x\_74 | x\_75 | x\_76 | x\_77 | x\_78 | cover | cover\_cumulative | rule\_no |
| --- | --- | --- | --- | --- | --- | --- | --- | --- | --- | --- | --- | --- | --- | --- | --- | --- | --- | --- | --- | --- | --- | --- | --- | --- | --- | --- | --- | --- | --- | --- | --- | --- | --- | --- | --- | --- | --- | --- | --- | --- | --- | --- | --- | --- | --- | --- | --- | --- | --- | --- | --- | --- | --- | --- | --- | --- | --- | --- | --- | --- | --- | --- | --- | --- | --- | --- | --- | --- | --- | --- | --- | --- | --- | --- | --- | --- | --- | --- | --- | --- | --- |
| 1.00 | when | CD123 | < | 1 |  |  | & | CD45 | >= | 2 | & | CD24 | < | 121 |  |  |  |  |  |  |  |  |  |  |  |  |  |  | & | CD10 | < | 121 |  |  | & | NG2 | is | 0 |  |  |  |  |  |  | & | CD22 | >= |  |  | 3 |  |  |  |  |  |  | & | CD81 | < | 12 |  |  |  |  |  |  |  |  |  |  |  |  |  |  |  |  |  |  | 8% | 8% | 1 |
| 0.00 | when | CD123 | >= |  |  | 1 | & | CD45 | < | 4 | & | CD24 | is | 15 | to | 84 | & | CD66c | < | 32 |  |  | & | TdT | < | 14 |  |  | & | CD10 | is | 8 | to | 33 |  |  |  |  | & | CD9 | is | 3 | to | 25 |  |  |  |  |  |  |  |  |  |  |  |  |  |  |  |  |  |  |  |  |  |  | & | CD38 | < | 7 |  |  |  |  |  |  |  |  | 6% | 14% | 2 |
| 1.00 | when | CD123 | < | 1 |  |  | & | CD45 | >= | 2 | & | CD24 | < | 106 |  |  | & | CD66c | < | 20 |  |  | & | TdT | is | 4 | to | 8 |  |  |  |  |  |  | & | NG2 | is | 0 |  |  |  |  |  |  | & | CD22 | < | 3 |  |  | & | CD34 | < | 3 |  |  |  |  |  |  |  |  |  |  |  |  |  |  |  |  |  |  |  |  |  |  |  |  | 5% | 19% | 3 |
| 0.59 | when | CD123 | < | 1 |  |  | & | CD45 | < | 2 | & | CD24 | >= |  |  | 26 | & | CD66c | < | 1 |  |  | & | TdT | < | 6 |  |  | & | CD10 | is | 23 | to | 73 | & | NG2 | is | 0 |  |  |  |  |  |  |  |  |  |  |  |  |  |  |  |  |  |  | & | CD81 | < | 4 |  |  |  |  |  |  |  |  |  |  |  |  |  |  |  |  |  |  | 4% | 23% | 4 |
| 0.82 | when | CD123 | < | 1 |  |  | & | CD45 | >= | 2 | & | CD24 | < | 106 |  |  | & | CD66c | < | 20 |  |  | & | TdT | is | 4 | to | 9 | & | CD10 | < | 70 |  |  | & | NG2 | is | 0 |  |  |  |  |  |  | & | CD22 | < | 3 |  |  | & | CD34 | is | 3 | to | 11 |  |  |  |  |  |  |  |  |  |  |  |  |  |  |  |  |  |  |  |  |  |  | 4% | 27% | 5 |
| 0.36 | when | CD123 | < | 1 |  |  | & | CD45 | < | 2 | & | CD24 | < | 26 |  |  | & | CD66c | < | 1 |  |  | & | TdT | < | 6 |  |  | & | CD10 | is | 23 | to | 73 | & | NG2 | is | 0 |  |  |  |  |  |  |  |  |  |  |  |  |  |  |  |  |  |  | & | CD81 | < | 4 |  |  |  |  |  |  |  |  |  |  |  |  |  |  |  |  |  |  | 3% | 30% | 6 |
| 0.67 | when | CD123 | < | 1 |  |  | & | CD45 | >= | 2 | & | CD24 | >= |  |  | 38 | & | CD66c | < | 20 |  |  | & | TdT | < | 4 |  |  |  |  |  |  |  |  | & | NG2 | is | 0 |  |  |  |  |  |  | & | CD22 | is | 1 | to | 3 | & | CD34 | < | 11 |  |  |  |  |  |  |  |  | & | CD13 | >= | 2 |  |  |  |  |  |  |  |  |  |  |  |  | 3% | 33% | 7 |
| 1.00 | when | CD123 | >= |  |  | 1 | & | CD45 | < | 4 | & | CD24 | < | 15 |  |  | & | CD66c | >= |  |  | 2 |  |  |  |  |  |  |  |  |  |  |  |  |  |  |  |  | & | CD9 | >= |  |  | 4 | & | CD22 | >= |  |  | 3 | & | CD34 | is | 2 | to | 10 |  |  |  |  |  |  |  |  |  |  |  |  |  |  |  |  |  |  |  |  |  |  | 3% | 36% | 8 |
| 1.00 | when | CD123 | is | 1 | to | 2 | & | CD45 | < | 4 | & | CD24 | >= |  |  | 5 |  |  |  |  |  |  |  |  |  |  |  |  |  |  |  |  |  |  |  |  |  |  | & | CD9 | < | 3 |  |  | & | CD22 | >= |  |  | 2 |  |  |  |  |  |  |  |  |  |  |  |  |  |  |  |  |  |  |  |  | & | CD33 | < | 2 |  |  |  |  | 3% | 39% | 9 |
| 1.00 | when | CD123 | < | 1 |  |  | & | CD45 | < | 2 | & | CD24 | >= |  |  | 4 |  |  |  |  |  |  | & | TdT | >= |  |  | 6 |  |  |  |  |  |  | & | NG2 | is | 0 |  |  |  |  |  |  | & | CD22 | >= |  |  | 3 |  |  |  |  |  |  | & | CD81 | < | 7 |  |  |  |  |  |  |  |  |  |  |  |  |  |  |  |  |  |  | 3% | 42% | 10 |
| 1.00 | when | CD123 | < | 1 |  |  | & | CD45 | < | 2 |  |  |  |  |  |  |  |  |  |  |  |  |  |  |  |  |  |  | & | CD10 | < | 63 |  |  | & | NG2 | is | 0 |  |  |  |  |  |  |  |  |  |  |  |  |  |  |  |  |  |  | & | CD81 | >= |  |  | 7 |  |  |  |  |  |  |  |  |  |  |  |  |  |  |  |  | 3% | 45% | 11 |
| 0.00 | when | CD123 | >= |  |  | 1 | & | CD45 | < | 4 | & | CD24 | is | 15 | to | 84 | & | CD66c | >= |  |  | 32 |  |  |  |  |  |  | & | CD10 | >= |  |  | 2 |  |  |  |  | & | CD9 | >= |  |  | 3 |  |  |  |  |  |  |  |  |  |  |  |  |  |  |  |  |  |  |  |  |  |  |  |  |  |  |  |  |  |  |  |  |  |  | 2% | 47% | 12 |
| 0.00 | when | CD123 | is | 1 | to | 2 | & | CD45 | < | 4 | & | CD24 | is | 15 | to | 29 | & | CD66c | < | 32 |  |  |  |  |  |  |  |  | & | CD10 | >= |  |  | 33 |  |  |  |  | & | CD9 | >= |  |  | 3 |  |  |  |  |  |  |  |  |  |  |  |  |  |  |  |  |  |  | & | CD13 | < | 3 |  |  |  |  |  |  |  |  |  |  |  |  | 2% | 49% | 13 |
| 0.00 | when | CD123 | < | 1 |  |  |  |  |  |  | & | CD24 | < | 6 |  |  |  |  |  |  |  |  |  |  |  |  |  |  |  |  |  |  |  |  | & | NG2 | is | 1 |  |  |  |  |  |  |  |  |  |  |  |  |  |  |  |  |  |  |  |  |  |  |  |  |  |  |  |  |  |  |  |  |  |  |  |  |  |  |  |  | 2% | 51% | 14 |
| 0.00 | when | CD123 | < | 1 |  |  | & | CD45 | < | 2 |  |  |  |  |  |  |  |  |  |  |  |  | & | TdT | < | 6 |  |  | & | CD10 | >= |  |  | 73 | & | NG2 | is | 0 |  |  |  |  |  |  |  |  |  |  |  |  |  |  |  |  |  |  | & | CD81 | < | 7 |  |  |  |  |  |  |  |  |  |  |  |  |  |  |  |  |  |  | 2% | 53% | 15 |
| 1.00 | when | CD123 | >= |  |  | 1 | & | CD45 | >= | 4 |  |  |  |  |  |  |  |  |  |  |  |  | & | TdT | >= |  |  | 3 |  |  |  |  |  |  |  |  |  |  |  |  |  |  |  |  |  |  |  |  |  |  |  |  |  |  |  |  |  |  |  |  |  |  |  |  |  |  |  |  |  |  |  |  |  |  |  |  |  |  | 2% | 55% | 16 |
| 1.00 | when | CD123 | < | 1 |  |  | & | CD45 | >= | 2 | & | CD24 | < | 22 |  |  | & | CD66c | < | 20 |  |  | & | TdT | < | 4 |  |  |  |  |  |  |  |  | & | NG2 | is | 0 |  |  |  |  |  |  | & | CD22 | is | 1 | to | 3 | & | CD34 | < | 11 |  |  |  |  |  |  |  |  | & | CD13 | >= | 2 |  |  |  |  |  |  |  |  |  |  |  |  | 2% | 57% | 17 |
| 1.00 | when | CD123 | < | 1 |  |  | & | CD45 | >= | 2 |  |  |  |  |  |  | & | CD66c | < | 20 |  |  |  |  |  |  |  |  |  |  |  |  |  |  | & | NG2 | is | 0 |  |  |  |  |  |  | & | CD22 | < | 3 |  |  | & | CD34 | >= |  |  | 12 |  |  |  |  |  |  |  |  |  |  |  |  |  |  |  |  |  |  |  |  |  |  | 2% | 59% | 18 |
| 0.00 | when | CD123 | >= |  |  | 1 | & | CD45 | < | 4 | & | CD24 | is | 15 | to | 84 | & | CD66c | < | 32 |  |  |  |  |  |  |  |  | & | CD10 | >= |  |  | 33 |  |  |  |  | & | CD9 | >= |  |  | 3 |  |  |  |  |  |  |  |  |  |  |  |  |  |  |  |  |  |  | & | CD13 | >= | 3 |  |  |  |  |  |  |  |  |  |  |  |  | 1% | 60% | 19 |
| 0.00 | when | CD123 | >= |  |  | 1 | & | CD45 | < | 4 | & | CD24 | is | 15 | to | 22 | & | CD66c | < | 32 |  |  |  |  |  |  |  |  | & | CD10 | < | 8 |  |  |  |  |  |  | & | CD9 | >= |  |  | 3 |  |  |  |  |  |  |  |  |  |  |  |  |  |  |  |  |  |  |  |  |  |  |  |  |  |  |  |  |  |  |  |  |  |  | 1% | 61% | 20 |
| 0.00 | when | CD123 | is | 1 | to | 3 | & | CD45 | < | 4 | & | CD24 | < | 15 |  |  | & | CD66c | < | 33 |  |  | & | TdT | >= |  |  | 2 |  |  |  |  |  |  |  |  |  |  | & | CD9 | >= |  |  | 3 |  |  |  |  |  |  | & | CD34 | >= |  |  | 10 |  |  |  |  |  |  |  |  |  |  |  |  |  |  |  |  |  |  |  |  |  |  | 1% | 62% | 21 |
| 0.00 | when | CD123 | is | 1 | to | 3 | & | CD45 | < | 4 | & | CD24 | < | 15 |  |  | & | CD66c | >= |  |  | 33 | & | TdT | >= |  |  | 2 |  |  |  |  |  |  |  |  |  |  | & | CD9 | >= |  |  | 3 | & | CD22 | >= |  |  | 3 | & | CD34 | >= |  |  | 10 |  |  |  |  |  |  |  |  |  |  |  |  |  |  |  |  |  |  |  |  |  |  | 1% | 63% | 22 |
| 0.00 | when | CD123 | >= |  |  | 2 | & | CD45 | < | 4 |  |  |  |  |  |  |  |  |  |  |  |  |  |  |  |  |  |  | & | CD10 | >= |  |  | 8 |  |  |  |  | & | CD9 | < | 3 |  |  |  |  |  |  |  |  |  |  |  |  |  |  |  |  |  |  |  |  | & | CD13 | < | 3 |  |  |  |  |  |  |  |  |  |  |  |  | 1% | 64% | 23 |
| 0.00 | when | CD123 | < | 1 |  |  | & | CD45 | < | 2 |  |  |  |  |  |  |  |  |  |  |  |  | & | TdT | < | 6 |  |  | & | CD10 | < | 3 |  |  | & | NG2 | is | 0 |  |  |  |  |  |  |  |  |  |  |  |  |  |  |  |  |  |  | & | CD81 | < | 7 |  |  |  |  |  |  |  |  |  |  |  |  |  |  |  |  |  |  | 1% | 65% | 24 |
| 0.00 | when | CD123 | < | 1 |  |  | & | CD45 | < | 2 |  |  |  |  |  |  |  |  |  |  |  |  | & | TdT | < | 6 |  |  | & | CD10 | is | 18 | to | 23 | & | NG2 | is | 0 |  |  |  |  |  |  |  |  |  |  |  |  |  |  |  |  |  |  | & | CD81 | < | 7 |  |  |  |  |  |  |  |  |  |  |  |  |  |  |  |  |  |  | 1% | 66% | 25 |
| 0.00 | when | CD123 | < | 1 |  |  | & | CD45 | < | 2 |  |  |  |  |  |  | & | CD66c | < | 1 |  |  | & | TdT | < | 6 |  |  | & | CD10 | is | 23 | to | 73 | & | NG2 | is | 0 |  |  |  |  |  |  |  |  |  |  |  |  |  |  |  |  |  |  | & | CD81 | is | 4 | to | 7 |  |  |  |  |  |  |  |  |  |  |  |  |  |  |  |  | 1% | 67% | 26 |
| 0.00 | when | CD123 | < | 1 |  |  | & | CD45 | < | 2 | & | CD24 | >= |  |  | 4 |  |  |  |  |  |  | & | TdT | >= |  |  | 6 | & | CD10 | is | 19 | to | 43 | & | NG2 | is | 0 |  |  |  |  |  |  | & | CD22 | < | 3 |  |  | & | CD34 | < | 5 |  |  | & | CD81 | < | 7 |  |  |  |  |  |  |  |  |  |  |  |  |  |  |  |  |  |  | 1% | 68% | 27 |
| 0.00 | when | CD123 | < | 1 |  |  | & | CD45 | >= | 2 |  |  |  |  |  |  | & | CD66c | >= |  |  | 20 |  |  |  |  |  |  |  |  |  |  |  |  | & | NG2 | is | 0 |  |  |  |  |  |  | & | CD22 | < | 3 |  |  |  |  |  |  |  |  |  |  |  |  |  |  |  |  |  |  |  |  |  |  |  |  |  |  |  |  |  |  | 1% | 69% | 28 |
| 0.00 | when | CD123 | < | 1 |  |  | & | CD45 | >= | 2 |  |  |  |  |  |  | & | CD66c | < | 20 |  |  | & | TdT | < | 4 |  |  |  |  |  |  |  |  | & | NG2 | is | 0 |  |  |  |  |  |  | & | CD22 | < | 3 |  |  | & | CD34 | < | 2 |  |  |  |  |  |  |  |  | & | CD13 | < | 2 |  |  |  |  |  |  |  |  |  |  |  |  | 1% | 70% | 29 |
| 0.20 | when | CD123 | >= |  |  | 1 | & | CD45 | < | 4 | & | CD24 | < | 15 |  |  |  |  |  |  |  |  |  |  |  |  |  |  |  |  |  |  |  |  |  |  |  |  | & | CD9 | >= |  |  | 3 |  |  |  |  |  |  | & | CD34 | < | 2 |  |  |  |  |  |  |  |  |  |  |  |  |  |  |  |  |  |  |  |  |  |  |  |  | 1% | 71% | 30 |
| 0.20 | when | CD123 | < | 1 |  |  | & | CD45 | >= | 2 | & | CD24 | is | 22 | to | 38 | & | CD66c | < | 20 |  |  | & | TdT | < | 4 |  |  |  |  |  |  |  |  | & | NG2 | is | 0 |  |  |  |  |  |  | & | CD22 | is | 1 | to | 3 | & | CD34 | < | 11 |  |  |  |  |  |  |  |  | & | CD13 | >= | 2 |  |  |  |  |  |  |  |  |  |  |  |  | 1% | 72% | 31 |
| 0.25 | when | CD123 | is | 1 | to | 2 | & | CD45 | < | 4 | & | CD24 | is | 29 | to | 84 | & | CD66c | < | 32 |  |  | & | TdT | < | 9 |  |  | & | CD10 | >= |  |  | 33 |  |  |  |  | & | CD9 | >= |  |  | 3 |  |  |  |  |  |  |  |  |  |  |  |  |  |  |  |  |  |  | & | CD13 | < | 3 |  |  |  |  |  |  |  |  |  |  |  |  | 1% | 73% | 32 |
| 0.25 | when | CD123 | >= |  |  | 2 | & | CD45 | < | 4 | & | CD24 | is | 15 | to | 84 | & | CD66c | < | 17 |  |  |  |  |  |  |  |  | & | CD10 | >= |  |  | 33 |  |  |  |  | & | CD9 | >= |  |  | 3 |  |  |  |  |  |  | & | CD34 | >= |  |  | 6 |  |  |  |  |  |  | & | CD13 | < | 3 |  |  |  |  |  |  |  |  |  |  |  |  | 1% | 74% | 33 |
| 0.33 | when | CD123 | >= |  |  | 1 | & | CD45 | < | 4 | & | CD24 | < | 15 |  |  | & | CD66c | >= |  |  | 2 |  |  |  |  |  |  |  |  |  |  |  |  |  |  |  |  | & | CD9 | >= |  |  | 3 | & | CD22 | < | 3 |  |  | & | CD34 | is | 2 | to | 10 |  |  |  |  |  |  |  |  |  |  |  |  |  |  |  |  |  |  |  |  |  |  | 1% | 75% | 34 |
| 0.33 | when | CD123 | < | 1 |  |  | & | CD45 | < | 2 |  |  |  |  |  |  |  |  |  |  |  |  | & | TdT | < | 6 |  |  | & | CD10 | is | 3 | to | 18 | & | NG2 | is | 0 |  |  |  |  |  |  |  |  |  |  |  |  | & | CD34 | is | 5 | to | 12 | & | CD81 | < | 7 |  |  |  |  |  |  |  |  |  |  |  |  |  |  |  |  |  |  | 1% | 76% | 35 |
| 0.33 | when | CD123 | < | 1 |  |  | & | CD45 | < | 2 |  |  |  |  |  |  |  |  |  |  |  |  |  |  |  |  |  |  | & | CD10 | >= |  |  | 63 | & | NG2 | is | 0 |  |  |  |  |  |  |  |  |  |  |  |  |  |  |  |  |  |  | & | CD81 | >= |  |  | 7 |  |  |  |  |  |  |  |  |  |  |  |  |  |  |  |  | 1% | 77% | 36 |
| 0.33 | when | CD123 | < | 1 |  |  | & | CD45 | >= | 2 | & | CD24 | < | 106 |  |  | & | CD66c | < | 20 |  |  | & | TdT | >= |  |  | 9 |  |  |  |  |  |  | & | NG2 | is | 0 |  |  |  |  |  |  | & | CD22 | < | 3 |  |  | & | CD34 | < | 11 |  |  |  |  |  |  |  |  |  |  |  |  |  |  |  |  |  |  |  |  |  |  |  |  | 1% | 78% | 37 |
| 0.33 | when | CD123 | < | 1 |  |  | & | CD45 | >= | 2 | & | CD24 | < | 106 |  |  | & | CD66c | < | 20 |  |  | & | TdT | is | 4 | to | 9 | & | CD10 | >= |  |  | 70 | & | NG2 | is | 0 |  |  |  |  |  |  | & | CD22 | < | 3 |  |  | & | CD34 | is | 3 | to | 11 |  |  |  |  |  |  |  |  |  |  |  |  |  |  |  |  |  |  |  |  |  |  | 1% | 79% | 38 |
| 0.33 | when | CD123 | < | 1 |  |  | & | CD45 | >= | 2 |  |  |  |  |  |  |  |  |  |  |  |  |  |  |  |  |  |  |  |  |  |  |  |  | & | NG2 | is | 0 |  |  |  |  |  |  | & | CD22 | >= |  |  | 3 |  |  |  |  |  |  | & | CD81 | >= |  |  | 12 |  |  |  |  |  |  |  |  |  |  |  |  |  |  |  |  | 1% | 80% | 39 |
| 0.67 | when | CD123 | >= |  |  | 2 | & | CD45 | < | 4 | & | CD24 | is | 15 | to | 84 | & | CD66c | is | 17 | to | 32 |  |  |  |  |  |  | & | CD10 | >= |  |  | 33 |  |  |  |  | & | CD9 | >= |  |  | 3 |  |  |  |  |  |  | & | CD34 | >= |  |  | 6 |  |  |  |  |  |  | & | CD13 | < | 3 |  |  |  |  |  |  |  |  |  |  |  |  | 1% | 81% | 40 |
| 0.67 | when | CD123 | >= |  |  | 3 | & | CD45 | < | 4 | & | CD24 | is | 22 | to | 84 | & | CD66c | < | 32 |  |  |  |  |  |  |  |  | & | CD10 | < | 8 |  |  |  |  |  |  | & | CD9 | >= |  |  | 3 |  |  |  |  |  |  |  |  |  |  |  |  |  |  |  |  |  |  |  |  |  |  |  |  |  |  |  |  |  |  |  |  |  |  | 1% | 82% | 41 |
| 0.67 | when | CD123 | is | 1 | to | 2 | & | CD45 | < | 4 | & | CD24 | < | 5 |  |  |  |  |  |  |  |  |  |  |  |  |  |  |  |  |  |  |  |  |  |  |  |  | & | CD9 | < | 3 |  |  | & | CD22 | >= |  |  | 2 |  |  |  |  |  |  |  |  |  |  |  |  |  |  |  |  |  |  |  |  | & | CD33 | < | 2 |  |  |  |  | 1% | 83% | 42 |
| 0.75 | when | CD123 | < | 1 |  |  | & | CD45 | < | 2 | & | CD24 | >= |  |  | 4 |  |  |  |  |  |  | & | TdT | >= |  |  | 6 | & | CD10 | is | 19 | to | 43 | & | NG2 | is | 0 |  |  |  |  |  |  | & | CD22 | < | 3 |  |  | & | CD34 | >= |  |  | 5 | & | CD81 | < | 7 |  |  |  |  |  |  |  |  |  |  |  |  |  |  |  |  |  |  | 1% | 84% | 43 |
| 0.75 | when | CD123 | < | 1 |  |  | & | CD45 | >= | 2 |  |  |  |  |  |  | & | CD66c | < | 20 |  |  | & | TdT | < | 4 |  |  |  |  |  |  |  |  | & | NG2 | is | 0 |  |  |  |  |  |  | & | CD22 | < | 3 |  |  | & | CD34 | is | 2 | to | 11 |  |  |  |  |  |  | & | CD13 | < | 2 |  |  |  |  |  |  |  |  |  |  |  |  | 1% | 85% | 44 |
| 0.75 | when | CD123 | < | 1 |  |  | & | CD45 | >= | 2 | & | CD24 | >= |  |  | 121 |  |  |  |  |  |  |  |  |  |  |  |  | & | CD10 | < | 121 |  |  | & | NG2 | is | 0 |  |  |  |  |  |  | & | CD22 | >= |  |  | 3 |  |  |  |  |  |  | & | CD81 | < | 12 |  |  |  |  |  |  |  |  |  |  |  |  |  |  |  |  |  |  | 1% | 86% | 45 |
| 0.80 | when | CD123 | >= |  |  | 1 | & | CD45 | < | 4 | & | CD24 | >= |  |  | 84 |  |  |  |  |  |  |  |  |  |  |  |  |  |  |  |  |  |  |  |  |  |  | & | CD9 | >= |  |  | 3 |  |  |  |  |  |  |  |  |  |  |  |  |  |  |  |  |  |  |  |  |  |  |  |  |  |  |  |  |  |  |  |  |  |  | 1% | 87% | 46 |
| 1.00 | when | CD123 | >= |  |  | 2 | & | CD45 | < | 4 | & | CD24 | is | 15 | to | 84 | & | CD66c | < | 32 |  |  |  |  |  |  |  |  | & | CD10 | >= |  |  | 33 |  |  |  |  | & | CD9 | >= |  |  | 3 |  |  |  |  |  |  | & | CD34 | < | 6 |  |  |  |  |  |  |  |  | & | CD13 | < | 3 |  |  |  |  |  |  |  |  |  |  |  |  | 1% | 88% | 47 |
| 1.00 | when | CD123 | is | 1 | to | 3 | & | CD45 | < | 4 | & | CD24 | is | 22 | to | 84 | & | CD66c | < | 32 |  |  |  |  |  |  |  |  | & | CD10 | < | 8 |  |  |  |  |  |  | & | CD9 | >= |  |  | 3 |  |  |  |  |  |  |  |  |  |  |  |  |  |  |  |  |  |  |  |  |  |  |  |  |  |  |  |  |  |  |  |  |  |  | 1% | 89% | 48 |
| 1.00 | when | CD123 | >= |  |  | 2 | & | CD45 | < | 4 |  |  |  |  |  |  |  |  |  |  |  |  |  |  |  |  |  |  | & | CD10 | < | 8 |  |  |  |  |  |  | & | CD9 | < | 3 |  |  |  |  |  |  |  |  |  |  |  |  |  |  |  |  |  |  |  |  |  |  |  |  |  |  |  |  |  |  |  |  |  |  |  |  | 1% | 90% | 49 |
| 1.00 | when | CD123 | < | 1 |  |  | & | CD45 | < | 2 |  |  |  |  |  |  | & | CD66c | >= |  |  | 1 | & | TdT | < | 6 |  |  | & | CD10 | is | 23 | to | 73 | & | NG2 | is | 0 |  |  |  |  |  |  |  |  |  |  |  |  |  |  |  |  |  |  | & | CD81 | < | 7 |  |  |  |  |  |  |  |  |  |  |  |  |  |  | & | CD20 | < | 6 | 1% | 91% | 50 |
| 1.00 | when | CD123 | < | 1 |  |  | & | CD45 | < | 2 |  |  |  |  |  |  |  |  |  |  |  |  | & | TdT | < | 6 |  |  | & | CD10 | is | 3 | to | 18 | & | NG2 | is | 0 |  |  |  |  |  |  |  |  |  |  |  |  | & | CD34 | < | 5 |  |  | & | CD81 | < | 7 |  |  |  |  |  |  |  |  |  |  |  |  |  |  |  |  |  |  | 1% | 92% | 51 |
| 1.00 | when | CD123 | < | 1 |  |  | & | CD45 | < | 2 |  |  |  |  |  |  |  |  |  |  |  |  | & | TdT | < | 6 |  |  | & | CD10 | is | 3 | to | 18 | & | NG2 | is | 0 |  |  |  |  |  |  |  |  |  |  |  |  | & | CD34 | >= |  |  | 12 | & | CD81 | < | 7 |  |  |  |  |  |  |  |  |  |  |  |  |  |  |  |  |  |  | 1% | 93% | 52 |
| 1.00 | when | CD123 | < | 1 |  |  | & | CD45 | < | 2 | & | CD24 | >= |  |  | 4 |  |  |  |  |  |  | & | TdT | >= |  |  | 6 | & | CD10 | < | 19 |  |  | & | NG2 | is | 0 |  |  |  |  |  |  | & | CD22 | < | 3 |  |  |  |  |  |  |  |  | & | CD81 | < | 7 |  |  |  |  |  |  |  |  |  |  |  |  |  |  |  |  |  |  | 1% | 94% | 53 |
| 1.00 | when | CD123 | < | 1 |  |  | & | CD45 | < | 2 | & | CD24 | >= |  |  | 4 |  |  |  |  |  |  | & | TdT | is | 6 | to | 12 | & | CD10 | >= |  |  | 43 | & | NG2 | is | 0 |  |  |  |  |  |  | & | CD22 | < | 3 |  |  |  |  |  |  |  |  | & | CD81 | < | 7 |  |  |  |  |  |  |  |  |  |  |  |  |  |  |  |  |  |  | 1% | 95% | 54 |
| 0.00 | when | CD123 | >= |  |  | 1 | & | CD45 | < | 4 | & | CD24 | < | 15 |  |  | & | CD66c | < | 2 |  |  |  |  |  |  |  |  |  |  |  |  |  |  |  |  |  |  | & | CD9 | >= |  |  | 3 |  |  |  |  |  |  | & | CD34 | is | 2 | to | 10 |  |  |  |  |  |  |  |  |  |  |  |  |  |  |  |  |  |  |  |  |  |  | 0% | 95% | 55 |
| 0.00 | when | CD123 | is | 1 | to | 2 | & | CD45 | < | 4 |  |  |  |  |  |  |  |  |  |  |  |  |  |  |  |  |  |  |  |  |  |  |  |  |  |  |  |  | & | CD9 | < | 3 |  |  | & | CD22 | < | 2 |  |  |  |  |  |  |  |  |  |  |  |  |  |  |  |  |  |  |  |  |  |  |  |  |  |  |  |  |  |  | 0% | 95% | 56 |
| 0.00 | when | CD123 | is | 1 | to | 2 | & | CD45 | < | 4 |  |  |  |  |  |  |  |  |  |  |  |  |  |  |  |  |  |  |  |  |  |  |  |  |  |  |  |  | & | CD9 | < | 3 |  |  | & | CD22 | >= |  |  | 2 |  |  |  |  |  |  |  |  |  |  |  |  |  |  |  |  |  |  |  |  | & | CD33 | >= | 2 |  |  |  |  | 0% | 95% | 57 |
| 0.00 | when | CD123 | >= |  |  | 1 | & | CD45 | >= | 4 |  |  |  |  |  |  |  |  |  |  |  |  | & | TdT | < | 3 |  |  |  |  |  |  |  |  |  |  |  |  |  |  |  |  |  |  |  |  |  |  |  |  |  |  |  |  |  |  |  |  |  |  |  |  |  |  |  |  |  |  |  |  |  |  |  |  |  |  |  |  | 0% | 95% | 58 |
| 0.00 | when | CD123 | < | 1 |  |  | & | CD45 | < | 2 | & | CD24 | < | 4 |  |  |  |  |  |  |  |  | & | TdT | >= |  |  | 6 |  |  |  |  |  |  | & | NG2 | is | 0 |  |  |  |  |  |  |  |  |  |  |  |  |  |  |  |  |  |  | & | CD81 | < | 7 |  |  |  |  |  |  |  |  |  |  |  |  |  |  |  |  |  |  | 0% | 95% | 59 |
| 0.00 | when | CD123 | < | 1 |  |  | & | CD45 | >= | 2 |  |  |  |  |  |  | & | CD66c | < | 20 |  |  |  |  |  |  |  |  |  |  |  |  |  |  | & | NG2 | is | 0 |  |  |  |  |  |  | & | CD22 | < | 3 |  |  | & | CD34 | is | 11 | to | 12 |  |  |  |  |  |  |  |  |  |  |  |  |  |  |  |  |  |  |  |  |  |  | 0% | 95% | 60 |
| 0.00 | when | CD123 | < | 1 |  |  | & | CD45 | >= | 2 |  |  |  |  |  |  | & | CD66c | < | 20 |  |  | & | TdT | < | 4 |  |  |  |  |  |  |  |  | & | NG2 | is | 0 |  |  |  |  |  |  | & | CD22 | < | 1 |  |  | & | CD34 | < | 11 |  |  |  |  |  |  |  |  | & | CD13 | >= | 2 |  |  |  |  |  |  |  |  |  |  |  |  | 0% | 95% | 61 |
| 0.00 | when | CD123 | < | 1 |  |  | & | CD45 | >= | 2 | & | CD24 | >= |  |  | 106 | & | CD66c | < | 20 |  |  | & | TdT | >= |  |  | 4 |  |  |  |  |  |  | & | NG2 | is | 0 |  |  |  |  |  |  | & | CD22 | < | 3 |  |  | & | CD34 | < | 11 |  |  |  |  |  |  |  |  |  |  |  |  |  |  |  |  |  |  |  |  |  |  |  |  | 0% | 95% | 62 |
| 0.00 | when | CD123 | < | 1 |  |  | & | CD45 | >= | 2 |  |  |  |  |  |  |  |  |  |  |  |  |  |  |  |  |  |  | & | CD10 | >= |  |  | 121 | & | NG2 | is | 0 |  |  |  |  |  |  | & | CD22 | >= |  |  | 3 |  |  |  |  |  |  | & | CD81 | < | 12 |  |  |  |  |  |  |  |  |  |  |  |  |  |  |  |  |  |  | 0% | 95% | 63 |
| 0.50 | when | CD123 | >= |  |  | 1 | & | CD45 | < | 4 | & | CD24 | is | 15 | to | 84 | & | CD66c | < | 32 |  |  | & | TdT | < | 14 |  |  | & | CD10 | is | 8 | to | 33 |  |  |  |  | & | CD9 | >= |  |  | 25 |  |  |  |  |  |  |  |  |  |  |  |  |  |  |  |  |  |  |  |  |  |  | & | CD38 | < | 7 |  |  |  |  |  |  |  |  | 0% | 95% | 64 |
| 0.50 | when | CD123 | >= |  |  | 1 | & | CD45 | < | 4 | & | CD24 | is | 15 | to | 84 | & | CD66c | < | 32 |  |  | & | TdT | < | 14 |  |  | & | CD10 | is | 8 | to | 33 |  |  |  |  | & | CD9 | >= |  |  | 3 |  |  |  |  |  |  |  |  |  |  |  |  |  |  |  |  |  |  |  |  |  |  | & | CD38 | >= | 7 |  |  |  |  |  |  |  |  | 0% | 95% | 65 |
| 0.50 | when | CD123 | >= |  |  | 3 | & | CD45 | < | 4 | & | CD24 | < | 15 |  |  |  |  |  |  |  |  | & | TdT | >= |  |  | 2 |  |  |  |  |  |  |  |  |  |  | & | CD9 | >= |  |  | 3 |  |  |  |  |  |  | & | CD34 | >= |  |  | 10 |  |  |  |  |  |  |  |  |  |  |  |  |  |  |  |  |  |  |  |  |  |  | 0% | 95% | 66 |
| 0.50 | when | CD123 | >= |  |  | 1 | & | CD45 | < | 4 | & | CD24 | < | 15 |  |  | & | CD66c | >= |  |  | 2 |  |  |  |  |  |  |  |  |  |  |  |  |  |  |  |  | & | CD9 | is | 3 | to | 4 | & | CD22 | >= |  |  | 3 | & | CD34 | is | 2 | to | 10 |  |  |  |  |  |  |  |  |  |  |  |  |  |  |  |  |  |  |  |  |  |  | 0% | 95% | 67 |
| 0.50 | when | CD123 | < | 1 |  |  | & | CD45 | < | 2 |  |  |  |  |  |  | & | CD66c | >= |  |  | 1 | & | TdT | < | 6 |  |  | & | CD10 | is | 23 | to | 73 | & | NG2 | is | 0 |  |  |  |  |  |  |  |  |  |  |  |  |  |  |  |  |  |  | & | CD81 | < | 7 |  |  |  |  |  |  |  |  |  |  |  |  |  |  | & | CD20 | >= | 6 | 0% | 95% | 68 |
| 0.50 | when | CD123 | < | 1 |  |  | & | CD45 | < | 2 | & | CD24 | >= |  |  | 4 |  |  |  |  |  |  | & | TdT | >= |  |  | 12 | & | CD10 | >= |  |  | 43 | & | NG2 | is | 0 |  |  |  |  |  |  | & | CD22 | < | 3 |  |  |  |  |  |  |  |  | & | CD81 | < | 7 |  |  |  |  |  |  |  |  |  |  |  |  |  |  |  |  |  |  | 0% | 95% | 69 |
| 0.50 | when | CD123 | < | 1 |  |  | & | CD45 | >= | 2 | & | CD24 | < | 106 |  |  | & | CD66c | < | 20 |  |  | & | TdT | is | 8 | to | 9 |  |  |  |  |  |  | & | NG2 | is | 0 |  |  |  |  |  |  | & | CD22 | < | 3 |  |  | & | CD34 | < | 3 |  |  |  |  |  |  |  |  |  |  |  |  |  |  |  |  |  |  |  |  |  |  |  |  | 0% | 95% | 70 |
| 1.00 | when | CD123 | >= |  |  | 1 | & | CD45 | < | 4 | & | CD24 | is | 15 | to | 84 | & | CD66c | >= |  |  | 32 |  |  |  |  |  |  | & | CD10 | < | 2 |  |  |  |  |  |  | & | CD9 | >= |  |  | 3 |  |  |  |  |  |  |  |  |  |  |  |  |  |  |  |  |  |  |  |  |  |  |  |  |  |  |  |  |  |  |  |  |  |  | 0% | 95% | 71 |
| 1.00 | when | CD123 | >= |  |  | 1 | & | CD45 | < | 4 | & | CD24 | is | 15 | to | 84 | & | CD66c | < | 32 |  |  | & | TdT | >= |  |  | 14 | & | CD10 | is | 8 | to | 33 |  |  |  |  | & | CD9 | >= |  |  | 3 |  |  |  |  |  |  |  |  |  |  |  |  |  |  |  |  |  |  |  |  |  |  |  |  |  |  |  |  |  |  |  |  |  |  | 0% | 95% | 72 |
| 1.00 | when | CD123 | is | 1 | to | 2 | & | CD45 | < | 4 | & | CD24 | is | 29 | to | 84 | & | CD66c | < | 32 |  |  | & | TdT | >= |  |  | 9 | & | CD10 | >= |  |  | 33 |  |  |  |  | & | CD9 | >= |  |  | 3 |  |  |  |  |  |  |  |  |  |  |  |  |  |  |  |  |  |  | & | CD13 | < | 3 |  |  |  |  |  |  |  |  |  |  |  |  | 0% | 95% | 73 |
| 1.00 | when | CD123 | is | 1 | to | 3 | & | CD45 | < | 4 | & | CD24 | < | 15 |  |  | & | CD66c | >= |  |  | 33 | & | TdT | >= |  |  | 2 |  |  |  |  |  |  |  |  |  |  | & | CD9 | >= |  |  | 3 | & | CD22 | < | 3 |  |  | & | CD34 | >= |  |  | 10 |  |  |  |  |  |  |  |  |  |  |  |  |  |  |  |  |  |  |  |  |  |  | 0% | 95% | 74 |
| 1.00 | when | CD123 | >= |  |  | 1 | & | CD45 | < | 4 | & | CD24 | < | 15 |  |  |  |  |  |  |  |  | & | TdT | < | 2 |  |  |  |  |  |  |  |  |  |  |  |  | & | CD9 | >= |  |  | 3 |  |  |  |  |  |  | & | CD34 | >= |  |  | 10 |  |  |  |  |  |  |  |  |  |  |  |  |  |  |  |  |  |  |  |  |  |  | 0% | 95% | 75 |
| 1.00 | when | CD123 | >= |  |  | 2 | & | CD45 | < | 4 |  |  |  |  |  |  |  |  |  |  |  |  |  |  |  |  |  |  | & | CD10 | >= |  |  | 8 |  |  |  |  | & | CD9 | < | 3 |  |  |  |  |  |  |  |  |  |  |  |  |  |  |  |  |  |  |  |  | & | CD13 | >= | 3 |  |  |  |  |  |  |  |  |  |  |  |  | 0% | 95% | 76 |
| 1.00 | when | CD123 | < | 1 |  |  |  |  |  |  | & | CD24 | >= |  |  | 6 |  |  |  |  |  |  |  |  |  |  |  |  |  |  |  |  |  |  | & | NG2 | is | 1 |  |  |  |  |  |  |  |  |  |  |  |  |  |  |  |  |  |  |  |  |  |  |  |  |  |  |  |  |  |  |  |  |  |  |  |  |  |  |  |  | 0% | 95% | 77 |

#### t(12;21)/ETV6-RUNX1 vs. hyperdiploidy

```
Confusion Matrix and Statistics

          Reference
Prediction   0   1
         0 119  10
         1   2  42
                                         
               Accuracy : 0.9306         
                 95% CI : (0.882, 0.9636)
    No Information Rate : 0.6994         
    P-Value [Acc > NIR] : 6.696e-14      
                                         
                  Kappa : 0.8275         
                                         
 Mcnemar's Test P-Value : 0.04331        
                                         
            Sensitivity : 0.8077         
            Specificity : 0.9835         
         Pos Pred Value : 0.9545         
         Neg Pred Value : 0.9225         
              Precision : 0.9545         
                 Recall : 0.8077         
                     F1 : 0.8750         
             Prevalence : 0.3006         
         Detection Rate : 0.2428         
   Detection Prevalence : 0.2543         
      Balanced Accuracy : 0.8956         
                                         
       'Positive' Class : 1
```

|  | Variable importance |
| --- | --- |
| CD66c | 20.357166 |
| CD10 | 19.495063 |
| CD24 | 9.285274 |
| TdT | 8.652694 |
| CD123 | 7.567173 |
| CD34 | 6.610682 |
| CD45 | 5.955695 |
| CD38 | 4.563971 |
| CD81 | 4.367285 |
| CD33 | 4.130608 |
| CD13 | 3.222222 |
| CD22 | 3.204678 |
| CyIgM | 2.951897 |

| probability: primary class | x | x\_2 | x\_3 | x\_4 | x\_5 | x\_6 | x\_7 | x\_8 | x\_9 | x\_10 | x\_11 | x\_12 | x\_13 | x\_14 | x\_15 | x\_16 | x\_17 | x\_18 | x\_19 | x\_20 | x\_21 | x\_22 | x\_23 | x\_24 | x\_25 | x\_26 | x\_27 | x\_28 | x\_29 | x\_30 | x\_31 | x\_32 | x\_33 | x\_34 | x\_35 | x\_36 | x\_37 | x\_38 | x\_39 | x\_40 | x\_41 | x\_42 | x\_43 | x\_44 | x\_45 | x\_46 | cover | cover\_cumulative | rule\_no |
| --- | --- | --- | --- | --- | --- | --- | --- | --- | --- | --- | --- | --- | --- | --- | --- | --- | --- | --- | --- | --- | --- | --- | --- | --- | --- | --- | --- | --- | --- | --- | --- | --- | --- | --- | --- | --- | --- | --- | --- | --- | --- | --- | --- | --- | --- | --- | --- | --- | --- |
| 0.03 | when | CD66c | >= | 1 | & | CD10 | < | 98 |  |  | & | CD24 | < | 36 | & | CD123 | >= | 1 |  |  |  |  |  |  |  |  | & | CD45 | < | 4 |  |  |  |  |  |  |  |  |  |  |  |  |  |  |  |  | 43% | 43% | 1 |
| 1.00 | when | CD66c | < | 1 | & | CD10 | >= |  |  | 31 |  |  |  |  |  |  |  |  |  |  |  |  | & | CD13 | < | 14 |  |  |  |  |  |  |  |  | & | CD38 | >= | 1 |  |  |  |  |  |  |  |  | 10% | 53% | 2 |
| 0.12 | when | CD66c | >= | 1 | & | CD10 | < | 98 |  |  | & | CD24 | >= | 36 | & | CD123 | >= | 1 |  |  |  |  |  |  |  |  |  |  |  |  |  |  |  |  |  |  |  |  | & | CD22 | >= | 3 |  |  |  |  | 9% | 62% | 3 |
| 0.09 | when | CD66c | >= | 1 |  |  |  |  |  |  | & | CD24 | < | 53 | & | CD123 | < | 1 | & | CD34 | >= | 2 | & | CD13 | < | 2 |  |  |  |  |  |  |  |  |  |  |  |  |  |  |  |  |  |  |  |  | 6% | 68% | 4 |
| 0.00 | when | CD66c | < | 1 | & | CD10 | is | 9 | to | 31 | & | CD24 | >= | 12 |  |  |  |  | & | CD34 | >= | 6 |  |  |  |  |  |  |  |  |  |  |  |  |  |  |  |  |  |  |  |  |  |  |  |  | 5% | 73% | 5 |
| 0.50 | when | CD66c | >= | 1 | & | CD10 | < | 98 |  |  | & | CD24 | >= | 36 | & | CD123 | >= | 1 |  |  |  |  |  |  |  |  |  |  |  |  |  |  |  |  |  |  |  |  | & | CD22 | < | 3 |  |  |  |  | 5% | 78% | 6 |
| 1.00 | when | CD66c | >= | 1 |  |  |  |  |  |  |  |  |  |  | & | CD123 | < | 1 | & | CD34 | < | 2 |  |  |  |  |  |  |  |  |  |  |  |  |  |  |  |  |  |  |  |  | & | CD81 | < | 5 | 4% | 82% | 7 |
| 0.25 | when | CD66c | < | 1 | & | CD10 | < | 31 |  |  | & | CD24 | >= | 12 |  |  |  |  | & | CD34 | < | 6 |  |  |  |  |  |  |  |  | & | TdT | < | 4 |  |  |  |  |  |  |  |  |  |  |  |  | 2% | 84% | 8 |
| 0.67 | when | CD66c | >= | 1 |  |  |  |  |  |  | & | CD24 | >= | 53 | & | CD123 | < | 1 | & | CD34 | >= | 2 | & | CD13 | < | 2 |  |  |  |  |  |  |  |  |  |  |  |  |  |  |  |  |  |  |  |  | 2% | 86% | 9 |
| 0.67 | when | CD66c | < | 1 | & | CD10 | >= |  |  | 31 |  |  |  |  |  |  |  |  |  |  |  |  | & | CD13 | < | 14 |  |  |  |  |  |  |  |  | & | CD38 | < | 1 |  |  |  |  |  |  |  |  | 2% | 88% | 10 |
| 1.00 | when | CD66c | < | 1 | & | CD10 | < | 31 |  |  | & | CD24 | >= | 12 |  |  |  |  | & | CD34 | < | 6 |  |  |  |  |  |  |  |  | & | TdT | >= | 4 |  |  |  |  |  |  |  |  |  |  |  |  | 2% | 90% | 11 |
| 1.00 | when | CD66c | < | 1 | & | CD10 | < | 31 |  |  | & | CD24 | < | 12 |  |  |  |  |  |  |  |  |  |  |  |  |  |  |  |  | & | TdT | < | 6 |  |  |  |  |  |  |  |  |  |  |  |  | 2% | 92% | 12 |
| 0.00 | when | CD66c | >= | 1 |  |  |  |  |  |  |  |  |  |  | & | CD123 | < | 1 | & | CD34 | >= | 2 | & | CD13 | >= | 2 | & | CD45 | >= | 2 |  |  |  |  |  |  |  |  |  |  |  |  |  |  |  |  | 1% | 93% | 13 |
| 0.00 | when | CD66c | >= | 1 |  |  |  |  |  |  |  |  |  |  | & | CD123 | < | 1 | & | CD34 | < | 2 |  |  |  |  |  |  |  |  |  |  |  |  |  |  |  |  |  |  |  |  | & | CD81 | >= | 5 | 1% | 94% | 14 |
| 0.00 | when | CD66c | < | 1 | & | CD10 | < | 31 |  |  | & | CD24 | < | 12 |  |  |  |  |  |  |  |  |  |  |  |  |  |  |  |  | & | TdT | >= | 6 |  |  |  |  |  |  |  |  |  |  |  |  | 1% | 95% | 15 |
| 0.00 | when | CD66c | < | 1 | & | CD10 | >= |  |  | 31 |  |  |  |  |  |  |  |  |  |  |  |  | & | CD13 | >= | 14 |  |  |  |  |  |  |  |  |  |  |  |  |  |  |  |  |  |  |  |  | 1% | 96% | 16 |
| 1.00 | when | CD66c | >= | 1 | & | CD10 | < | 98 |  |  | & | CD24 | < | 36 | & | CD123 | >= | 1 |  |  |  |  |  |  |  |  | & | CD45 | >= | 4 |  |  |  |  |  |  |  |  |  |  |  |  |  |  |  |  | 1% | 97% | 17 |
| 1.00 | when | CD66c | >= | 1 | & | CD10 | >= |  |  | 98 |  |  |  |  | & | CD123 | >= | 1 |  |  |  |  |  |  |  |  |  |  |  |  |  |  |  |  |  |  |  |  |  |  |  |  |  |  |  |  | 1% | 98% | 18 |
| 1.00 | when | CD66c | >= | 1 |  |  |  |  |  |  |  |  |  |  | & | CD123 | < | 1 | & | CD34 | >= | 2 | & | CD13 | >= | 2 | & | CD45 | < | 2 |  |  |  |  |  |  |  |  |  |  |  |  |  |  |  |  | 1% | 99% | 19 |
| 1.00 | when | CD66c | < | 1 | & | CD10 | < | 9 |  |  | & | CD24 | >= | 12 |  |  |  |  | & | CD34 | >= | 6 |  |  |  |  |  |  |  |  |  |  |  |  |  |  |  |  |  |  |  |  |  |  |  |  | 1% | 100% | 20 |
